# Unraveling of cocatalysts photodeposited selectively on facets of BiVO_4_ to boost solar water splitting

**DOI:** 10.1038/s41467-022-28146-6

**Published:** 2022-01-25

**Authors:** Yu Qi, Jiangwei Zhang, Yuan Kong, Yue Zhao, Shanshan Chen, Deng Li, Wei Liu, Yifan Chen, Tengfeng Xie, Junyan Cui, Can Li, Kazunari Domen, Fuxiang Zhang

**Affiliations:** 1 State Key Laboratory of Catalysis, Dalian Institute of Chemical Physics, Chinese Academy of Sciences, Dalian, 116023 China; 2 Hefei National Laboratory for Physical Sciences at the Microscale and Department of Chemical Physics, University of Science and Technology of China, Hefei, Anhui 230026 China; 3 Research Initiative for Supra-Materials (RISM), Shinshu University, 4-17-1 Wakasato, Nagano, 380-8553 Japan; 4 College of Chemistry, Jilin University, Changchun, Jilin 130012 China; 5 School of Material Science and Engineering, Zhengzhou University, No. 100 Science Avenue, Zhengzhou, 450001 China; 6 Office of University Professors, The University of Tokyo, 7-3-1 Hongo, Bunkyo-ku, Tokyo 113-8656 Japan

**Keywords:** Photocatalysis, Artificial photosynthesis, Nanoscale materials

## Abstract

Bismuth vanadate (BiVO_4_) has been widely investigated as a photocatalyst or photoanode for solar water splitting, but its activity is hindered by inefficient cocatalysts and limited understanding of the underlying mechanism. Here we demonstrate significantly enhanced water oxidation on the particulate BiVO_4_ photocatalyst via in situ facet-selective photodeposition of dual-cocatalysts that exist separately as metallic Ir nanoparticles and nanocomposite of FeOOH and CoOOH (denoted as FeCoO_x_), as revealed by advanced techniques. The mechanism of water oxidation promoted by the dual-cocatalysts is experimentally and theoretically unraveled, and mainly ascribed to the synergistic effect of the spatially separated dual-cocatalysts (Ir, FeCoO_x_) on both interface charge separation and surface catalysis. Combined with the H_2_-evolving photocatalysts, we finally construct a Z-scheme overall water splitting system using [Fe(CN)_6_]^3−/4−^ as the redox mediator, whose apparent quantum efficiency at 420 nm and solar-to-hydrogen conversion efficiency are optimized to be 12.3% and 0.6%, respectively.

## Abstract (web summary)

Artificial photosynthesis offers an integrated means to convert light to fuel, but efficiencies are often low. Here, authors report a Z-scheme system utilizing Ir and FeCoO_x_ co-catalysts to enhance charge separation on BiVO_4_ facets that achieves high quantum efficiencies for overall water splitting.

## Introduction

Particulate photocatalytic overall water splitting (OWS) based on inorganic semiconductor materials with relative good photostability has been demonstrated as one of the most promising ways of realizing scalable and economically viable solar hydrogen production to address energy- and environment-related issues^1–12^. To achieve high solar-to-hydrogen (STH) energy conversion efficiency, it is necessary to increase the quantum efficiency of photocatalytic OWS over a wide range of wavelengths, particularly the use of visible light^11,12^. However, extended visible light utilization is generally accompanied by a decreased driving force of the photogenerated carriers to make charge separation extremely difficult. Furthermore, the construction of OWS systems faces serious challenges originating from the sluggish kinetics of water oxidation involving uphill energy barrier and multiple electron transfer^13^. Consequently, visible-light-driven photocatalytic OWS systems are not only limited in number, but also show lower efficiency than those driven by ultraviolet light^14–19^. Accordingly, it is highly desirable to precisely design and modify photocatalysts with efficient visible light utilization for promotion of water oxidation.

**Fig. 1:**
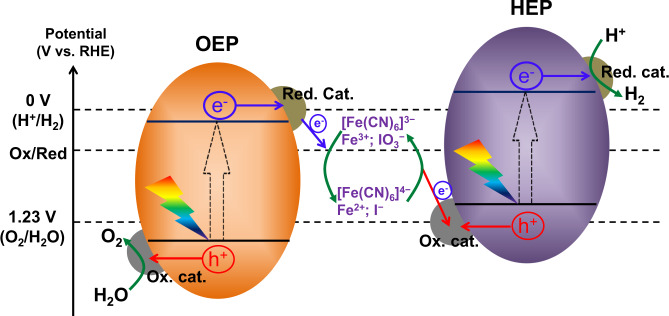
The energy diagram for a two-step photoexcitation (also called Z-scheme) system with an aqueous redox mediator for overall water splitting. Red. cat.: reduction cocatalyst; O_x_. cat.: oxidation cocatalyst; RHE: reversible hydrogen electrode; HEP: H_2_-evolving photocatalyst; OEP: O_2_-evolving photocatalyst.

N-type monoclinic bismuth vanadate (BiVO_4_) has emerged as one of the most promising visible-light-responsive photocatalysts and photoanodes for water oxidation since Kudo’s report in 1998^20–28^. Owing to its advantages, such as efficient light absorption in the visible light region, good carrier mobility, controllable exposed facets, and non-toxic properties, BiVO_4_ semiconductor has been widely and successfully employed as the water oxidation photocatalyst for the assembly of Z-scheme OWS systems using solid conductor (i.e., Au, reduced graphene oxide) or redox couple (i.e., Fe^3+/2+^, [Fe(CN)_6_]^3−/4−^) as electron mediator^15,16,29–31^. Specifically, our previous work revealed that the spatial separation of photogenerated electrons and holes can be achieved on the anisotropic facets of BiVO_4_^32^, based on which reduction and oxidation cocatalysts are selectively deposited on different facets to remarkably promote its water oxidation and the efficiency of OWS under visible light^16^. Although the BiVO_4_ photocatalyst has been widely investigated for the assembly of artificial Z-scheme OWS systems, the apparent quantum efficiency (AQE) and STH conversion efficiency achieved so far are still considerably below what is expected. This is mainly due to the shortage of effective cocatalyst regulation and the lack of in-depth understanding of the microscopic mechanisms behind it^33,34^. Notably, for the assembly of the redox couple-mediated Z-scheme OWS system shown in Fig. 1, the loading of effective cocatalysts is extremely important not only for the acceleration of interfacial electron transfer between the H_2_-evolving photocatalyst (HEP) and the O_2_-evolving photocatalyst (OEP), but also for the promotion of surface reaction kinetics of water splitting^35–38^. Therefore, it is a long-term task to develop innovative cocatalysts and unravel their structures as well as influence mechanism on water splitting.

In this study, we address the sluggish water oxidation of BiVO_4_ via in situ photodeposition of dual innovative cocatalysts, with emphasis on elucidating the local structures of the cocatalysts and the mechanism of promotion of water oxidation. We demonstrate that the nanocomposite of FeOOH and CoOOH (denoted as FeCoO_x_) in situ formed on the {110} facet of BiVO_4_ not only lowers Gibbs free energy barrier of water oxidation, but also makes a better promotion on the electron transfer as well as charge separation compared with the commonly used CoO_x_ cocatalyst. Furthermore, the Ir cocatalyst in situ deposited on the {010} facet of BiVO_4_ was found to exhibit superior reduction ability of [Fe(CN)_6_]^3−^ ions to our previously reported Au. Based on the facet-selective loading of the innovative dual-cocatalysts, the evolution rate of O_2_ on the BiVO_4_ was significantly enhanced, and a particulate Z-scheme OWS system with an AQE of 12.3% at 420 nm and a STH of 0.6%, was finally fabricated using [Fe(CN)_6_]^3−/4−^ as a redox mediator and ZrO_2_/TaON or MgTa_2_O_6−x_N_y_/TaON as the HEP. Our results demonstrate the importance and effectiveness of developing suitable cocatalysts for enhancing interfacial charge separation and surface water oxidation kinetics in promoting solar energy conversion.

## Results

### Structural characterizations of cocatalysts photodeposited

**Fig. 2:**
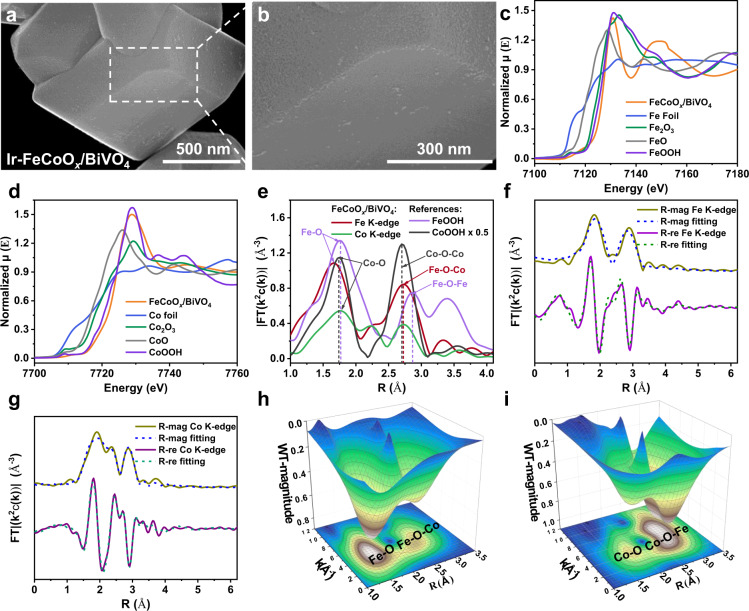
Morphology and structural characterizations of typical samples. **a**, **b** FESEM images of the Ir-FeCoO_x_/BiVO_4_ with different magnification times. **c**–**i** Structural characterizations of the FeCoO_x_/BiVO_4_ sample together with references: **c** Normalized Fe K-edge XANES μ(E) spectra. **d** Normalized Co K-edge XANES μ(E) spectra. **e** Fe K-edge and Co K-edge radial distance χ(R) space spectra. **f** Fourier-transformed (FT)-Extended X-ray absorption fine structure (EXAFS) fitting curves at R space of Fe K-edge. **g** FT-EXAFS fitting curves at R space of Co K-edge. **h** Fe K-edge 3D contour wavelet transform. **i** Co K-edge 3D contour wavelet transform.

The anisotropic BiVO_4_ with exposed {010} and {110} facets was prepared according to our previous study^39^. The Ir nanoparticles and FeCoO_x_ nanocomposite were in situ photodeposited on the surface of BiVO_4_ from an aqueous solution containing the precursors K_2_IrCl_6_, CoSO_4_, and redox [Fe(CN)_6_]^3−^ ions. The as-obtained sample is hereafter denoted as Ir-FeCoO_x_/BiVO_4_. As expected from our previous findings on the spatial separation of photogenerated electrons and holes on the anisotropic BiVO_4_^32^, the Ir nanoparticles, and FeCoO_x_ nanocomposite were clearly observed to be selectively deposited on the {010} and {110} facets of BiVO_4_, respectively (Fig. 2a, b). For comparison, the in situ photodeposition of single Ir or CoO_x_ particles on BiVO_4_ was similarly obtained (denoted as Ir/BiVO_4_ and CoO_x_/BiVO_4_), and the sample was characterized by field-emission scanning electron microscopy (FESEM) to further confirm the facet-selective deposition (Supplementary Fig. 1). It should be noted that the morphology of the cocatalysts located on the {110} facet of Ir-FeCoO_x_/BiVO_4_ (Fig. 2b) is clearly different from that of the CoO_x_/BiVO_4_ sample (Supplementary Fig. 1b), demonstrating the possible interaction between Fe and Co-based compounds. And the change in the long wavelength range of UV-Vis diffuse reflectance spectra (DRS) can confirm the successful deposition of the dual-cocatalysts (Supplementary Fig. 2). The deposited Ir species were verified to exist as metallic Ir nanoparticles by means of X-ray absorption near edge structure (XANES) spectroscopy (Supplementary Fig. 3) and high-resolution transmission electron microscopy image (Supplementary Fig. 4).

To unravel the formation of the FeCoO_x_ nanocomposite on the {110} facets of BiVO_4_, the existing state and dispersion of both Fe and Co elements on FeCoO_x_/BiVO_4_ (free of Ir nanoparticles to rule out its possible interference during characterization) were first analyzed. According to the elemental mapping results shown in Supplementary Fig. 5, both Fe and Co species are similarly located and dispersed, accompanied by the existence of O, which further demonstrates that Co and Fe combine together in the form of oxidation state during the photo-oxidation process. The deposition of Fe should result from redox ions in the reaction solution. The coexistence of both Fe and Co can be further revealed by electron energy loss spectroscopy analysis (Supplementary Fig. 6). And their oxidation states can be confirmed to be Fe^3+^ and Co^3+^ by the Fe and Co K-edge XANES measurements through comparing with the reference materials (Fig. 2c, d, respectively).

Second, the radial distance space spectra χ(R) of Fe and Co in FeCoO_x_/BiVO_4_ and their corresponding references were analyzed, which provides more convincing support for the formation of nanocomposite. As shown in Fig. 2e and Supplementary Fig. 7, the peaks located at approximately 2.72 Å assigned to the Fe–O–Co bond are consistently observed in both the Fe and Co K-edge of the FeCoO_x_/BiVO_4_ sample, but no scattering path signals attributing to the Co–Co bond (2.41 Å) from Co foil, Fe–Fe bond (2.47 Å) from Fe foil, Co–O–Co bond (2.69 Å) from CoOOH, or Fe–O–Fe bond (2.86 Å) from FeOOH can be observed. This clearly reveals that the formation of nanocomposite is a homogeneous phase of bimetallic hydroxide, instead of single-phase Fe or Co hydroxides. It should be pointed out that the possible nanocomposite of Fe_2_O_3_ and Co_2_O_3_ can be ruled out by comparing the fingerprint feature pattern of normalized XANES μ(E) spectra (Fig. 2c, d and Supplementary Fig. 8a, b) and the first derivative of the normalized XANES μ(E) spectra (Supplementary Fig. 8c, d). In particular, as shown in Supplementary Fig. 8c, d, the peak positions of FeCoO_x_/BiVO_4_ are closer to the FeOOH and CoOOH references. Based on these results, the phase species of the FeCoO_x_ on the surface of BiVO_4_ sample can be deduced to be more similar to FeOOH/CoOOH with respect to Fe_2_O_3_/Co_2_O_3_. In addition, compared with the corresponding single-phase hydroxides FeOOH and CoOOH, Fe/BiVO_4_ sample exhibits much shorter Fe–O bond and longer Co–O bond, and the length of Co–O–Fe bond is between Co–O–Co and Fe–O–Fe (Fig. 2e). This demonstrates the existence of electron transfer and a strong interaction between Fe and Co in the FeCoO_x_/BiVO_4_ sample, providing further proof about the formation of the nanocomposite.

Third, the formation of the nanocomposite can be further verified by the results of quantitative χ(R) space spectra fitting and wavelet transform of χ(k). As seen in Supplementary Table 1, Fe–O–Co bond with similar coordination numbers (Fe–O–Co: 2 at ca. 2.745 Å in Fe K-edge; Co–O–Fe: 2 at ca. 2.761 Å in Co K-edge) can be confirmed. The good fitting results of χ(R) and χ(k) space spectra (Fig. 2f, g and Supplementary Fig. 9) with reasonable R-factors and the obtained fitting parameters (Supplementary Table 1) provide a quantitative illustration of the existence of Fe–O–Co bond originating from the nanocomposite structure. As similarly revealed in Fig. 2h, i, the Fe–O–Co bond located at [χ(k), χ(R)] of [4.2, 2.74] or Co–O–Fe bond ([6.4, 2.76]) as well as the Fe–O bond ([4.8, 1.64]) or Co–O bond ([4.2, 1.88]) with two scattering path signal can be observed for both Fe and Co K-edge wavelet transform of χ(k) spectra of FeCoO_x_/BiVO_4_, but the characteristic scattering path signal of Fe–Fe bond ([8.4, 2.52]), Co–Co bond ([7.8, 2.42]), Fe–O–Fe bond ([5.6 2.82]) or Co–O–Co bond ([6.8, 2.78]) is not observed as similarly as the reference sample (Supplementary Fig. 10).

### Effect of reduction and oxidation cocatalysts

**Fig. 3:**
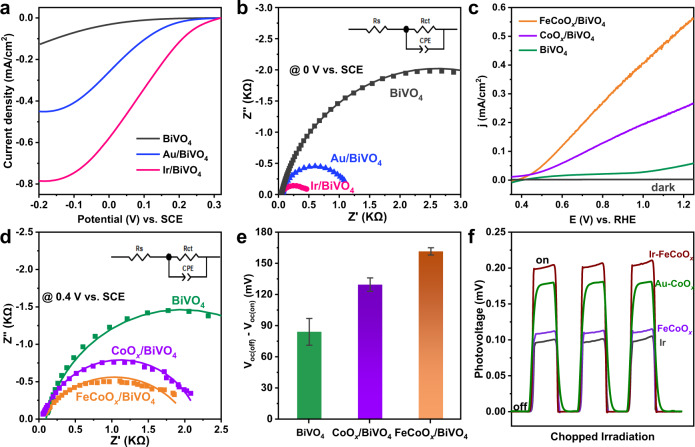
Electrochemical measurements and characterizations of typical samples. **a** Linear sweep voltammetry curves of typical samples in the 100 mM sodium phosphate buffer solution (pH 6.0) containing 5 mM K_3_[Fe(CN)_6_]. **b** EIS spectra of typical samples in a 100 mM sodium phosphate buffer solution (pH 6.0) containing 5 mM K_3_[Fe(CN)_6_]. SCE, saturated calomel electrode. **c** Photocurrent density-potential curves of BiVO_4_, CoO_x_/BiVO_4_, and FeCoO_x_/BiVO_4_. **d** EIS spectra of BiVO_4_, CoO_x_/BiVO_4_, and FeCoO_x_/BiVO_4_ in a 100 mM sodium phosphate buffer solution (pH 6.0). **e** Comparison of difference of OCPs on the BiVO_4_, CoO_x_/BiVO_4_, and FeCoO_x_/BiVO_4_ under dark and illumination conditions. Measurements were taken at least three times for separate samples and average values are presented with the standard deviation as the error bar. **f** Comparison of promotion effect of cocatalysts on the SPV values of the BiVO_4_ photocatalyst under chopped visible light irradiation.

As shown in Fig. 1, the water oxidation process of OEP is strongly dependent on both the reduction and oxidation cocatalysts. Therefore, understanding the effect of deposited Ir and FeCoO_x_ cocatalysts is highly desirable. As depicted in Fig. 3a, the ability of the deposited metallic Ir to reduce [Fe(CN)_6_]^3−^ ions was evaluated and found to exhibit a much higher cathode current than that of our previously reported Au nanoparticles on BiVO_4_^16^, indicating its superior performance in activating and reducing the [Fe(CN)_6_]^3−^ ions. In addition, the deposition of Ir or Au cocatalyst on the surface of BiVO_4_ can significantly decrease the charge-transfer resistance (R_ct_) across the semiconductor/electrolyte interface (Fig. 3b), further revealing the effectiveness of the deposited cocatalysts in accelerating the electron transfer from BiVO_4_ to the [Fe(CN)_6_]^3−^ ions (values of R_s_ and R_ct_ listed in Supplementary Table 2). Meanwhile, the promotion effect of Ir is better than that of Au.

To determine the effect of the FeCoO_x_ nanocomposite, the efficiencies of charge separation and injection (denoted as η_sep_ and η_inj_, respectively) on the FeCoO_x_/BiVO_4_ photoanode (CoO_x_/BiVO_4_ and BiVO_4_ as references) were evaluated by referring to a previous photoelectrochemical analysis^22^. Figure 3c and Supplementary Fig. 11a show that the current of the BiVO_4_ photoanode can be remarkably promoted after the deposition of FeCoO_x_ and CoO_x_ in both cases, with and without the use of a hole scavenger, among which FeCoO_x_ exhibits a much better promotion effect than CoO_x_. On this basis, both η_sep_ and η_inj_ on the FeCoO_x_/BiVO_4_ photoanode were calculated to be higher than that of the CoO_x_/BiVO_4_ photoanode (Supplementary Fig. 11c, d), demonstrating the better promotion effect of the FeCoO_x_ nanocomposite on both the separation of photogenerated carriers and the injection of holes into the reaction solution (i.e., surface reaction) with respect to CoO_x_. The excellent promotion of FeCoO_x_ on the surface reaction can be further supported by the electrochemical impedance spectroscopy (EIS) results given in Fig. 3d and Supplementary Table 3, based on which the R_ct_ resistance on the FeCoO_x_/BiVO_4_ electrode is the smallest among the three electrodes investigated. On the other hand, the superior promotion effect of FeCoO_x_ on the charge separation can also be evidenced by its larger open-circuit potential (OCP) on the FeCoO_x_/BiVO_4_ compared with CoO_x_/BiVO_4_ (Fig. 3e). Based on the previous result that a larger difference of OCPs under dark and illumination conditions corresponds to more intense band bending^40^, therefore the FeCoO_x_/BiVO_4_ sample can be deduced to own a more intense band bending than the CoO_x_/BiVO_4_ sample, leading to a significantly improved η_sep_ and the more intense band bending should result from the p-n heterojunction between FeCoO_x_ and BiVO_4_^41^.

Encouraged by the understanding of the functionalities of both reduction and oxidation cocatalysts (i.e., Ir and FeCoO_x_), the synergistic effect of dual-cocatalysts on the charge separation was examined using the surface photovoltage (SPV) spectrum. As shown in Fig. 3f, the sample with both Ir and FeCoO_x_ deposited exhibits a greater SPV amplitude with respect to the sample with single Ir or FeCoO_x_ loaded. It should be mentioned that a much better promotion effect is also observed for the sample with facet-selective deposition of Ir and FeCoO_x_ compared to that with facet-selective deposition of Au and CoO_x_. These results reveal the importance of both facet-selective deposition of dual-cocatalysts and the development of innovative cocatalysts for maximizing the promotion effect.

### Density functional theory calculations on the O_2_-evolving reaction

**Fig. 4:**
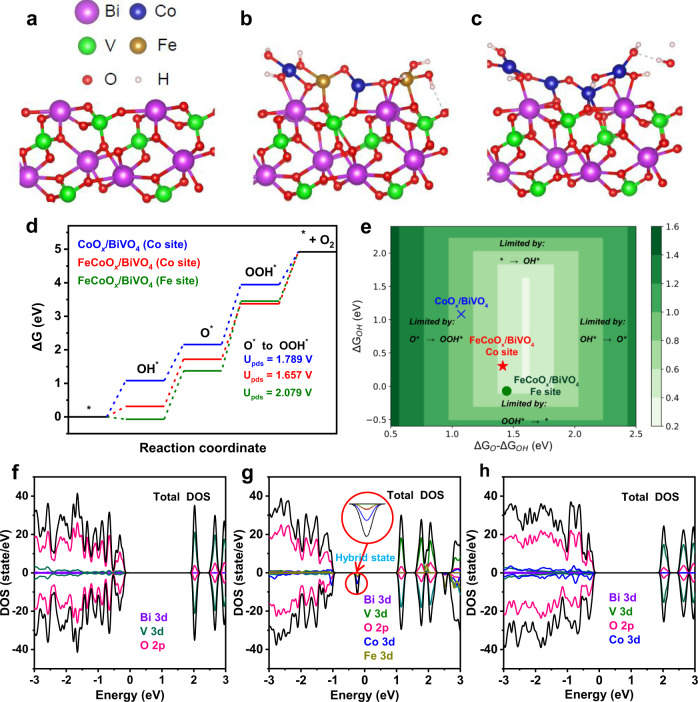
Theoretical understanding of the promotion effect of the FeCoO_x_ cocatalyst. Visual representation of structures of BiVO_4_ {110} surface (**a**) FeCoO_x_/BiVO_4_ {110} interface (**b**) and CoO_x_/BiVO_4_ {110} interface (**c**) for the DFT calculations. **d** Free energy diagram for OER process on FeCoO_x_/BiVO_4_ and CoO_x_/BiVO_4_ {110} interfaces. The surface structures with various reaction intermediates are shown alongside the free energy diagram. U_pds_, equilibrium potential for the potential determining step. **e** Theoretical overpotential plot with O^∗^ and OH^∗^ binding energies as descriptors. Calculated densities of state for the BiVO_4_ {110} surface (**f**), FeCoO_x_/BiVO_4_ {110} interface (**g**), and CoO_x_/BiVO_4_ {110} interface (**h**).

Density functional theory (DFT) calculations were performed to further elucidate the microscopic mechanism of the promotion effect of the FeCoO_x_ cocatalyst on the O_2_-evolving reaction (OER) from the viewpoint of both surface catalysis and interfacial charge transfer. As shown in Fig. 4a–c, the CoO_x_–FeO_x_–CoO_x_–FeO_x_ and CoO_x_–CoO_x_–CoO_x_–CoO_x_ clusters were simply extracted and placed on the {110} facets of BiVO_4_ to simulate the FeCoO_x_/BiVO_4_ and CoO_x_/BiVO_4_ interfaces, respectively, which origin from the structure of EXAFS measurement. And the schematic of the whole OER mechanism on the FeCoO_x_/BiVO_4_ and CoO_x_/BiVO_4_ is given in Supplementary Fig. 12 and illustrated in detail in supporting information. Fig. 4d and e presents the Gibbs free energy change diagram of the four elementary steps of OER on the surface of FeCoO_x_/BiVO_4_ and CoO_x_/BiVO_4_, during which the Co and Fe sites on FeCoO_x_/BiVO_4_ were mainly considered as the active sites, respectively. It was demonstrated that the rate-determining step of FeCoO_x_/BiVO_4_ (Co or Fe site) and CoO_x_/BiVO_4_ is the adsorption of one OH^−^ to form OOH^∗^ from O^∗^. The largest decrease of the Gibbs free energy barrier was observed for FeCoO_x_/BiVO_4_ (Co site), whereas the OER performance of FeCoO_x_/BiVO_4_ (Fe site) is much weaker than that of the corresponding Co site, suggesting that the Co site acts as the main OER site.

Next, we plotted the calculated densities of states (DOS) of the BiVO_4_ {110} surface, CoO_x_/BiVO_4_ {110} interface, and FeCoO_x_/BiVO_4_ {110} interface (Fig. 4f, g, h and Supplementary Fig. 13). Both CoO_x_ and FeCoO_x_ are set to be located at the {110} facets of BiVO_4_. For the bare BiVO_4_ {110} surface, there is a direct bandgap about 2.1 eV between the valence band and conduction band (Fig. 4f). However, when the CoO_x_–FeO_x_–CoO_x_–FeO_x_ cluster is settled on the {110} surface of BiVO_4_, a mixed band mainly composed of Co 3d, Fe 3d, and O 2p states emerges between the valence band and conduction band (Fig. 4g). It has been demonstrated that the localization of photoexcited holes, as well as subsequent charge separation can be promoted through the formation of mixed bands^42^. In addition, the DOS of the CoO_x_/BiVO_4_ {110} interface (Fig. 4h) has no similar result as that of the FeCoO_x_/BiVO_4_ {110} interface (Fig. 4g, bandgap = 2.0 eV), implying that the loading of FeCoO_x_ on BiVO_4_ should have better charge separation. In order to microscopically understand the better electron transfer on the FeCoO_x_ with respect to the CoO_x_, their bader charges were calculated and compared. As given in Supplementary Table 4, the changing trend of bader charge on the Co active site after introduction of Fe (increase from 1.2 a.u. in CoO_x_/BiVO_4_ to 1.3 a.u. in FeCoO_x_/BiVO_4_) is in line with the changing one of experimental valence state (Supplementary Fig. 14). Compared to the CoO_x_/BiVO_4_, the higher bader charge on the Co active site in FeCoO_x_/BiVO_4_ indicates its stronger oxidation capacity as well as more beneficial electron transfer^43^. In addition, as shown in Supplementary Fig. 15, the d-band center (Ed) value of Co active sites in FeCoO_x_/BiVO_4_ was calculated as −1.63 eV, which is sharply increased with respect to the CoO_x_/BiVO_4_ (−2.56 eV). This demonstrates that the electronic structure of Co active sites can be well modulated and optimized in the FeCoO_x_/BiVO_4_ due to the introduction of Fe atoms to get much stronger adsorption properties to the OER intermediates according to the d-band center theory^44,45^. According to previous experimental and theoretical demonstration, the Fe site is relatively inactive during the OER process^46,47^. So we deduce that the role of Fe is to assist in modifying the geometric and electronic structure of Co in the OER together with our results that very limited contributions of Fe 3d states are observed for the mixed band (Fig. 4g). These conclusions from DFT calculation well match with the aforementioned experimental results.

### Photocatalytic performances of Z-scheme OWS

**Fig. 5:**
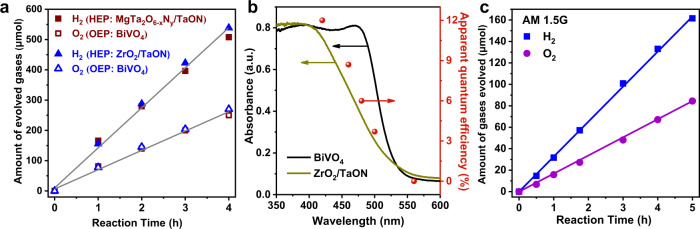
Photocatalytic activity of Z-scheme OWS. **a** Time course of Z-scheme OWS on the optimized conditions under visible light irradiation. **b** Dependence curve of AQE value as a function of irradiation wavelength, and UV-Vis DRS of the HEP and OEP. **c** Time curve of Z-scheme OWS under illumination of the standard solar simulator (AM 1.5 G, 100 mW cm^−2^). Reaction conditions: **a** 50 mg OEP, 50 mg HEP (ZrO_2_/TaON, 1.0 wt% Rh, 1.5 wt% Cr) or 50 mg OEP, 100 mg HEP (MgTa_2_O_6-x_N_y_/TaON, 2.5 wt% Rh, 3.75 wt% Cr), 100 mL 25 mM sodium phosphate buffer solution (pH 6.0) containing K_4_[Fe(CN)_6_] (10 mM), 300 W xenon lamp (λ ≥ 420 nm), temperature: 288 K, Pyrex top-irradiation type. **b** 75 mg OEP, 75 mg HEP (ZrO_2_/TaON, 1.0 wt% Rh, 1.5 wt% Cr), 150 mL 25 mM sodium phosphate buffer solution (pH 6.0) containing K_4_[Fe(CN)_6_] (10 mM), 300 W xenon lamp, temperature: 298 K, Pyrex top-irradiation type. (c) 50 mg OEP, 50 mg HEP (ZrO_2_/TaON, 1.0 wt% Rh, 1.5 wt% Cr), 100 mL 25 mM sodium phosphate buffer solution (pH 6.0) containing K_4_[Fe(CN)_6_] (10 mM), temperature: 288 K, Pyrex top-irradiation type.

The modified BiVO_4_ was employed as an OEP for the assembly of efficient Z-scheme OWS systems together with ZrO_2_/TaON or MgTa_2_O_6−x_N_y_/TaON as a HEP under visible light irradiation. The HEPs were prepared and modified with cocatalysts according to previously reported procedures^16,48,49^, and the diffraction structure and morphology features were coarsely revealed by their powder X-ray diffraction patterns and FESEM images (Supplementary Figs. 16, 17). The contents of deposited Ir and Co on BiVO_4_ were optimized to be 0.8 and 0.2 wt%, respectively, via the photocatalytic O_2_ evolution reaction (Supplementary Figs. 18, 19). As seen in Fig. 5a, stable evolution curves of H_2_ and O_2_ with the stoichiometric molar ratio of 2:1 can be observed at the experimental region using the optimized photocatalysts, indicating the successful achievement of the OWS process. Moreover, regardless of using ZrO_2_/TaON or MgTa_2_O_6−x_N_y_/TaON as the HEP, similar OWS activities with the initial rates of H_2_ and O_2_ evolution (ca. 160 and 80 μmol/h, respectively) were separately observed, implying that the O_2_ evolution on BiVO_4_ is the rate-determining step, as similarly observed in our previous study^16^. It should be pointed out that when the Ir-CoO_x_(Imp.)/BiVO_4_ with Ir and Co randomly impregnated is employed as the OEP, the OWS will not be achieved owing to the significantly decreased O_2_-evolving activity (Supplementary Fig. 20). This indicates the importance of facet-selective deposition of dual-cocatalysts in promoting the O_2_-evolving activity and fabricating a successful OWS system. The multiple cycles of time-course curves shown in Supplementary Figs. 21 and 22 demonstrate the good photostability of the system constructed in this study. The AQE value of OWS as a function of absorption wavelength was found to be in good accordance with the UV-Vis DRS of the OEP and HEP, indicating that the Z-scheme OWS system is driven by visible light excitation (Fig. 5b and Supplementary Fig. 23). The optimal AQE value of OWS at 420 ± 10 nm is 12.3%, and the AQE value at the 500 ± 10 nm is about 3%, demonstrating the wide visible light utilization. According to the activity measurements under the irradiation of AM 1.5 G (Fig. 5c), the STH energy conversion efficiency was calculated to be 0.6%. To the best of our knowledge, both the AQE and STH values should be the highest among the suspending particulate photocatalytic OWS systems using inorganic semiconductor materials with visible light utilization, regardless of one-step or two-step (i.e., Z-scheme) systems.

## Discussion

Here, we show a highly efficient Z-scheme OWS system with benchmarked AQE and STH value over particulate inorganic semiconductor photocatalysts with visible light utilization. The success is mainly ascribed to the in situ facet-selective photodeposition of innovative dual-cocatalysts (Ir nanoparticles and FeCoO_x_ nanocomposite), based on which the sluggish water oxidation on BiVO_4_ can be largely overcome. Besides the finding and structural unraveling of efficient cocatalysts, the microscopic work mechanism of both reduction and oxidation cocatalysts on the interfacial charge transfer and surface catalysis has been well elucidated respectively. These results should be encouraging and enlightening to the design and assembly of OWS systems for more efficient solar-to-chemical energy conversion.

## Methods

### Synthesis of modified-TaON and BiVO_4_

ZrO_2_-modified TaON (Zr/Ta = 0.1) sample and MgTa_2_O_6−x_N_y_/TaON (Mg/Ta = 0.15) composite were used as the HEPs. The ZrO_2_-modified sample was synthesized by nitridation of the ZrO_2_/Ta_2_O_5_ composite and the MgTa_2_O_6−x_N_y_/TaON was prepared by nitridation of the MgTa_2_O_6_/Ta_2_O_5_ composite under an ammonia flow (20 mL min^–1^) at 1123 K for 15 h by referring to the previous works^48,49^. BiVO_4_ was chosen as the OEP, which was similarly synthesized according to our previous hydrothermal process^39^. Typically, 10 mmol NH_4_VO_3_ and 10 mmol Bi(NO_3_)_3_·5H_2_O were dissolved in 2.0 M nitric acid solution, whose pH value was then adjusted to be about 0.5 with ammonia solution (25–28 wt%). The mixed solution was strongly stirred until the observation of a light yellow precipitate that was further aged for about 2 h and then transferred to a Teflon-lined stainless steel autoclave for 10 h hydrothermal treatment at 473 K.

### Preparation of Ir/BiVO_4_ and CoO_x_/BiVO_4_

The deposition of Ir or CoO_x_ on the surface of BiVO_4_ was carried out by the photodeposition method. Typically, 0.2 g BiVO_4_ powder was dispersed in deionized water containing a calculated amount of K_2_IrCl_6_ (2.0 wt%) or CoSO_4_ (2.0 wt%), and hole (CH_3_OH) or electron (NaIO_3_) scavenger, separately. The well-mixed solution was then irradiated by 300 W xenon lamp free of any cut-off filter for 2 h. The as-obtained powders after filtration and washing are correspondingly denoted as Ir/BiVO_4_ and CoO_x_/BiVO_4_, which were used for further characterizations and tests.

### Preparation of FeCoO_x_/BiVO_4_ and Ir-FeCoO_x_/BiVO_4_

Both of the samples were similarly prepared by the in situ photodeposition. Meanwhile, 25 mM phosphate buffer solution (PBS, pH = 6, 50 mL) containing a calculated amount of CoSO_4_ and [Fe(CN)_6_]^3−^ ions was prepared for the synthesis of FeCoO_x_/BiVO_4_, while 25 mM phosphate buffer solution (PBS, pH = 6, 50 mL) containing a calculated amount of K_2_IrCl_6_, CoSO_4_ and [Fe(CN)_6_]^3−^ ions was prepared for synthesis of Ir-FeCoO_x_/BiVO_4_.

### Preparation of HEP

The deposition of nanoparticulate rhodium-chromium mixed oxides (denoted as Rh_y_Cr_2−y_O_3_) as a cocatalyst was carried out by the photodeposition method. 0.2 g ZrO_2_-modified TaON or MgTa_2_O_6−x_N_y_/TaON was dispersed in 20 v% 150 mL methanol solution. A certain amount of Na_3_RhCl_6_ and K_2_CrO_4_ (1.0 wt% Rh and 1.5 wt% Cr vs. photocatalyst for ZrO_2_/TaON and 2.0 wt% Rh and 3.75 wt% Cr vs. photocatalyst for MgTa_2_O_6-x_N_y_/TaON) were added as the precursors. The deposition was carried out under the full-spectral irradiation of 300 W xenon lamp for 6 h. Whereafter, the irradiated solution was centrifuged and washed with distilled water, and then dried at 353 K for overnight to get powder for use.

### Preparation of BiVO_4_ electrodes

The BiVO_4_ photoanode was prepared according to the previous work^50^. First of all, Bi(NO_3_)_3_·5H_2_O, NH_4_VO_3_, and polyvinyl alcohol were dissolved in 60% HNO_3_ to prepare the precursor solution. Then the precursor solution was spin-coated on the FTO followed by heat treatment at 623 K for 2 h in air to form the BiVO_4_ seed layer. Second, the treated FTO was immersed in 2.0 M HNO_3_ aqueous solution containing Bi(NO_3_)_3_·5H_2_O and NH_4_VO_3_, whose pH was adjusted to be 0.9 by adding NH_3_·H_2_O drop by drop. The formed BiVO_4_ precursor film solution was transferred to a Teflon-lined autoclave with the as-prepared substrate for hydrothermal treatment at 473 K for 12 h. The BiVO_4_ photoanode film was finally calcined at 773 K for 4 h.

As for the selective deposition of Ir and FeCoO_x_ cocatalysts on the BiVO_4_ photoanode, similar in situ photodeposition method as the powder was adopted. Specifically, the photoanode was immersed in 25 mM phosphate buffer solution (PBS, pH = 6, 50 mL) containing the K_2_IrCl_6_ or/and CoSO_4_ (K_2_IrCl_6_: 40 μL; CoSO_4_: 10 μL, the concentration of solution: 1 mg/mL) and K_3_[Fe(CN)_6_] (0.5 mM) and irradiated for 3 h. Similarly, CoO_x_ was photodeposited on the surface of BiVO_4_ to prepare the CoO_x_/BiVO_4_ photoanode.

### Measurements of AQE and STH conversion efficiency

The AQE was measured using a Pyrex top-irradiation-type reaction vessel and a 300 W xenon lamp fitted with band-pass filters (ZBPA420, Asahi Spectra Co., FWHM: 10 nm). The number of photons reaching the solution was measured using a calibrated Si photodiode (LS-100, EKO Instruments Co., LTD.), and the AQE (ϕ) was calculated using the following Eq. (1):

1 $$\phi ( \% )=(AR/I)\,\times 100$$

where *A*, *R*, and *I* are coefficients, *A* represents a coefficient (4 for H_2_ evolution; 8 for O_2_ evolution) and *R* represents the evolution rate of H_2_ or O_2_. As measured and calculated, the total number of incident photons at the wavelength of 420, 460, 480, 500, and 560 nm are 8.4 × 10^20^, 6.5  × 10^20^, 7.1 × 10^20^, 4.8 × 10^20^, and 6.9 × 10^20^ photons h^−1^, respectively. The evolution rates of H_2_ on the system containing Rh_y_Cr_2–y_O_3_-ZrO_2_/TaON and Ir-FeCoO_x_/BiVO_4_ photocatalysts under the wavelength of 420, 460, 480, 500, and 560 nm were tested to be 41.6, 23.0, 17.5, 7.4, and 0 μmol h^−1^, respectively. The evolution rates of H_2_ on the system containing Rh_y_Cr_2–y_O_3_-MgTa_2_O_6–x_N_y_/TaON and Ir-FeCoO_x_/BiVO_4_ photocatalysts under the wavelength of 420, 460, 480, and 560 nm were tested to be 42.4, 19.0, 9.0, and 0 μmol h^−1^, respectively.

The STH energy conversion efficiency (*η*) was calculated according to the following Eq. (2):

2 $$\eta ( \% )=({R}_{{{{{{\mathrm{H}}}}}}}\times \varDelta {G}^{{\rm O}})/(P\times S)\,\times 100$$

where *R*_H_, Δ*G*°, *P*, and *S* denote the rate of H_2_ evolution (mol s^−1^) in photocatalytic water splitting, standard Gibbs energy of water (237.13 × 10^3 ^J mol^−1^), intensity of simulated sunlight (0.1 W cm^−2^), and irradiation area (4.0 cm^2^), respectively. The light source was an AM 1.5 G solar simulator (XES-40S2-CE, San-Ei Electric), and a top-irradiation reaction vessel was used. The initial rates of H_2_ and O_2_ evolution are about 36 and 18 μmol/h, separately.

### Photoelectrochemical tests

As for the tests of linear sweep voltammetry (LSV) and EIS, a platinum plate was used as a counter electrode and the saturated calomel electrode (SCE) as the reference electrode. The phosphate buffer solution (pH = 6, 0.1 M) with 5 mM K_3_[Fe(CN)_6_] aqueous solution and phosphate buffer solution (pH = 6, 0.1 M) were used as the electrolyte. The potential of the working electrode was controlled by a potentiostat (CHI 660E) for the LSV test and potentiostat (Solartron analytic AMETEK) for the EIS test. Before the measurement, the solution was purged with argon gas. The Nyquist plots calculated from EIS were performed from 100,000 to 0.1 Hz. Data were fitted using Zview software.

Current–voltage (*J*–*V*) curves under irradiation and darkness were recorded on an electrochemical workstation (CHI 660E). The OCP of photoanode were recorded under illumination and darkness using electrochemical workstation (Solartron analytic AMETEK). A 300 W xenon lamp was used as the light source and the irradiation intensity was high enough to produce a flat band condition of the photoanodes. The electrolyte for *J*–*V* curves and OCP was 1 M KBi (pH = 9). 0.2 M Na_2_SO_3_ was added to the electrolyte as a hole scavenger if necessary.

## Supplementary information

Supplementary Information

Peer Review File

## Data Availability

The data that support the findings of this study are available from the source data. Source data are provided with this paper.

## Source data

Source Data
