# Supplementary material for: Unraveling of cocatalysts photodeposited selectively on facets of BiVO4 to boost solar water splitting
Source: Nat Commun. 2022 Jan 25;13:484. doi: 10.1038/s41467-022-28146-6 (PMC8789891; doi:10.1038/s41467-022-28146-6)
Supplement: Supplementary file 1 — Supplementary Information [file 41467_2022_28146_MOESM1_ESM.pdf]

Supplementary Information

Unraveling of cocatalysts photodeposited selectively on facets of $BiVO_4$ to boost solar water splitting

Yu Qi[1#], Jiangwei Zhang[1#], Yuan Kong[2#], Yue Zhao[1], Shanshan Chen[3], Deng Li[1], Wei Liu[1], Yifan Chen[4], Tengfeng Xie[4], Junyan Cui[5], Can Li[1*], Kazunari Domen[3,6] and Fuxiang Zhang[1*]

[1] State Key Laboratory of Catalysis, Dalian Institute of Chemical Physics, Chinese Academy of Sciences, Dalian 116023, China

[2] Hefei National Laboratory for Physical Sciences at the Microscale and Department of Chemical Physics, University of Science and Technology of China, Hefei, Anhui 230026, China

[3] Research Initiative for Supra-Materials (RISM), Shinshu University, 4-17-1 Wakasato, Nagano 380-8553, Japan

[4] College of Chemistry, Jilin University, Changchun, Jilin 130012, China

[5] School of Material Science and Engineering, Zhengzhou University, No.100 Science Avenue, Zhengzhou 450001, China

[6] Office of University Professors, The University of Tokyo, 7-3-1 Hongo, Bunkyo-ku, Tokyo 113-8656, Japan

Supplementary Figures

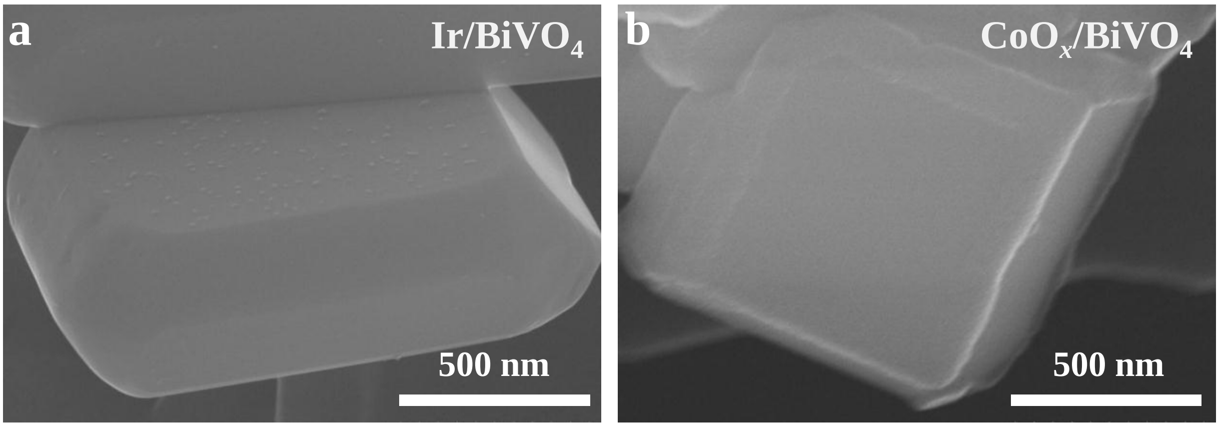

Supplementary Fig. 1 Typical FESEM images of the $BiVO_4$ loaded with different cocatalysts. (a) Ir/$BiVO_4$, (b) CoO_x/$BiVO_4$. The Ir nanoparticles are deposited on the {010} facet of $BiVO_4$ and the CoO_x is deposited on the {110} facet of $BiVO_4$.

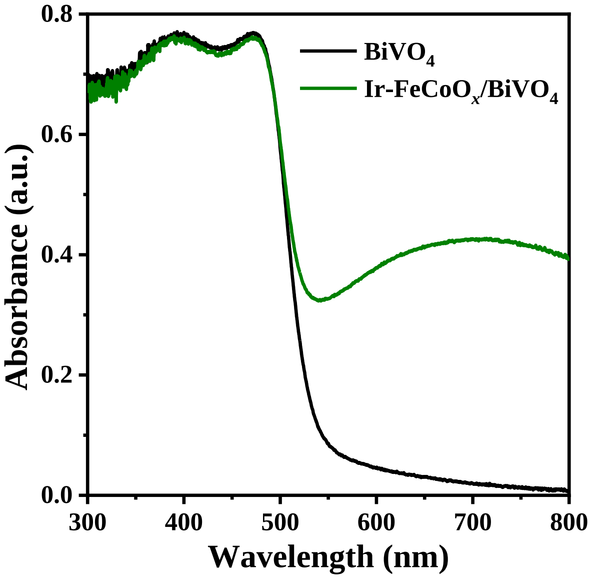

Supplementary Fig. 2 UV-vis DRS of the $BiVO_4$ and Ir-$FeCoO_x$/$BiVO_4$. The long wavelength absorption increases after loading the dual-cocatalysts, indicating the successful deposition of the dual-cocatalysts.

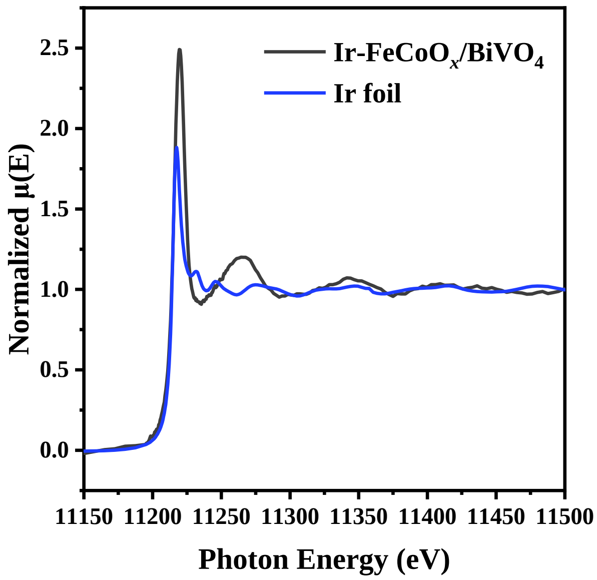

Supplementary Fig. 3 The XANES spectroscopy of Ir element of on the Ir-FeCoO$_x$/BiVO$_4$. The deposited Ir species are verified to exist as metallic Ir after comparing with the Ir foil as the reference.

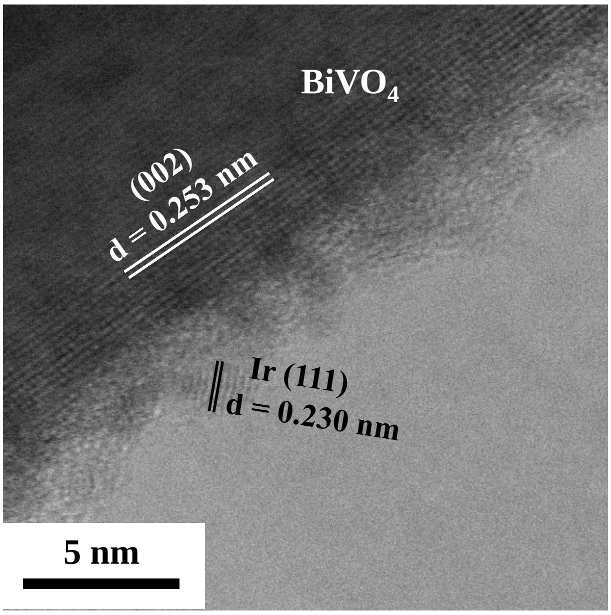

Supplementary Fig. 4 The Representative HRTEM image of Ir-FeCoO$_x$/BiVO$_4$. The lattice spacing of 0.230 nm is attributed to Ir and the nanoparticle size is about 2~3 nm.

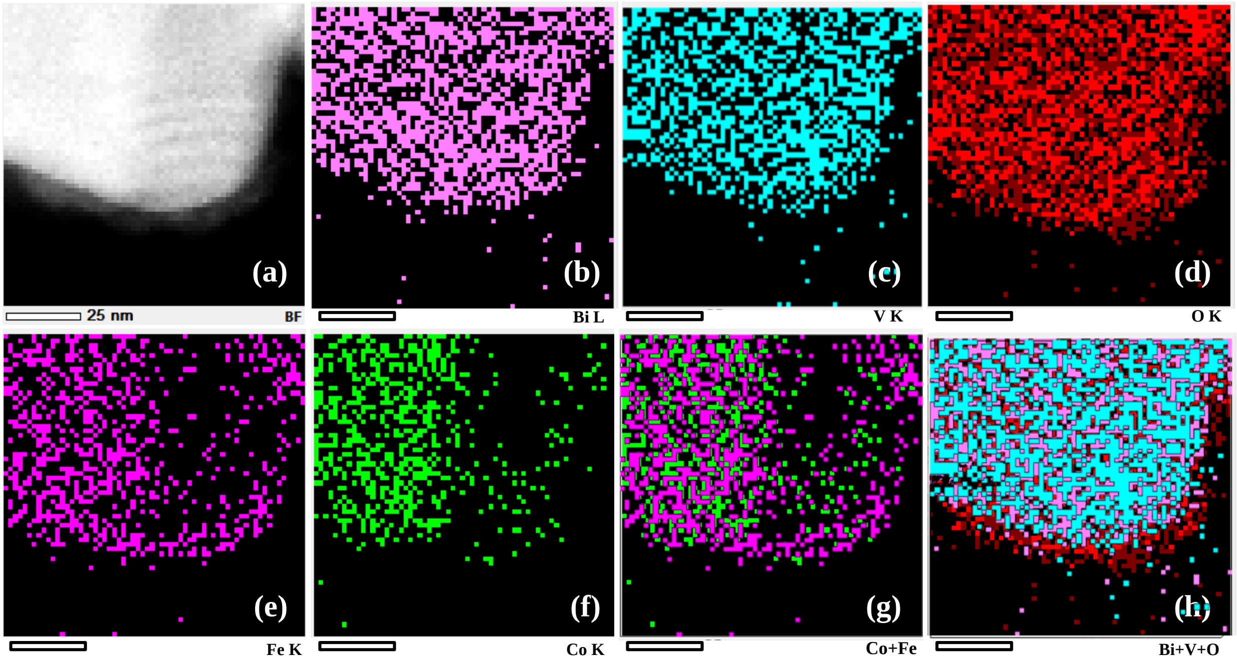

Supplementary Fig. 5 Elemental mappings of $FeCoO_x/BiVO_4$. (a) STEM image, (b) Bi element, (c) V element, (d) O element, (e) Fe element, (f) Co element, (g) simulated dispersion of Co and Fe element and (h) simulated dispersion of Bi, V and O element. The Fe, Co and O species are similarly located and dispersed, demonstrating that Co and Fe combine together in the form of oxidation state.

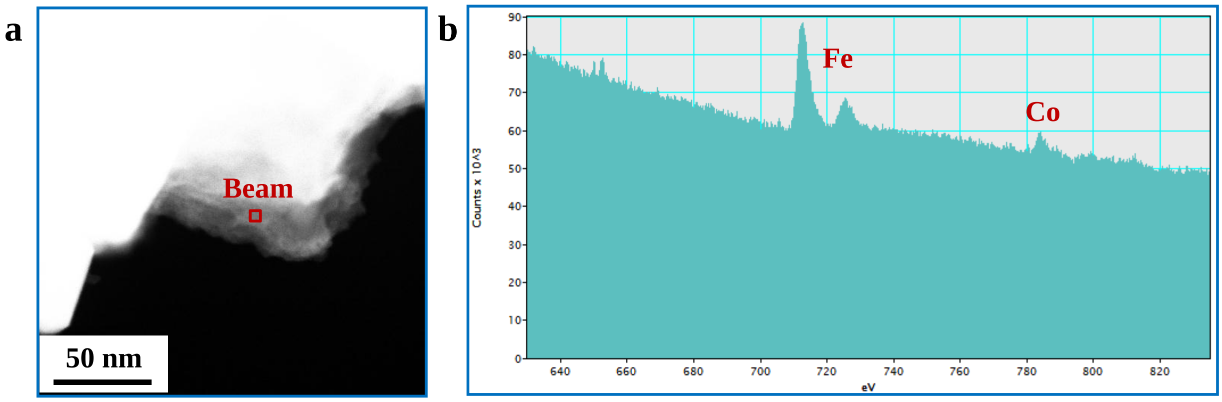

Supplementary Fig. 6 Confirmation of Fe and Co elements. (a) STEM image of $FeCoO_x/BiVO_4$ and (b) EELS spectra of the beam place. Both Fe and Co are simultaneously detected in the beam area.

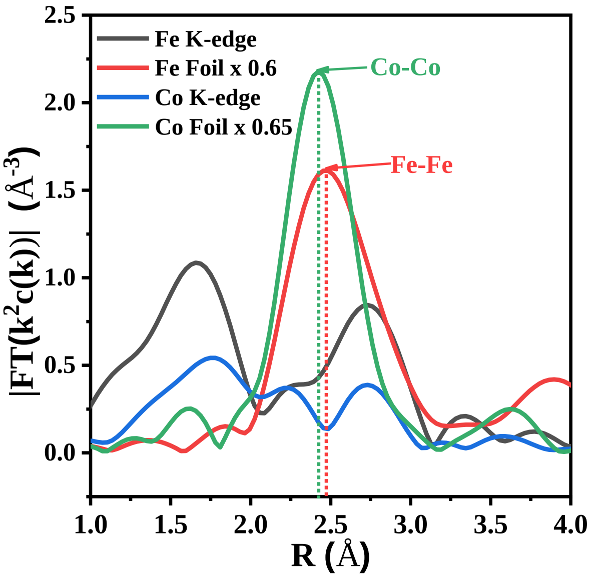

Supplementary Fig. 7 Radial distance χ(R) space spectra of $FeCoO_x/BiVO_4$. Fe K-edge and Fe foil as reference, Co K-edge and Co foil as reference. There is no Fe-Fe and Co-Co bond in the $FeCoO_x/BiVO_4$ sample.

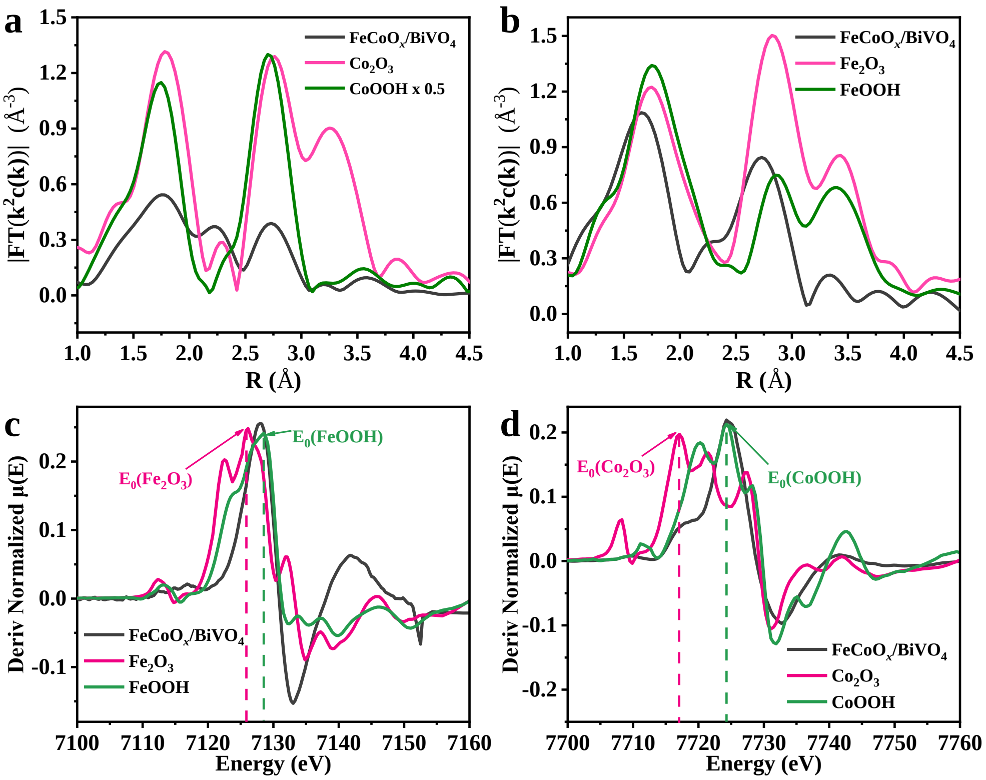

Supplementary Fig. 8 Comparison of fingerprint features of typical samples. (a,b) Radial distance χ(R) space spectra of $FeCoO_x/BiVO_4$: Fe K-edge and FeOOH, Fe_2O_3 as reference (a), Co K-edge and CoOOH, Co_2O_3 as reference (b); (c,d) The first derivative of Normalized XANES μ(E) spectra of $FeCoO_x/BiVO_4$: Fe K-edge and FeOOH, Fe_2O_3 as reference (c), Co K-edge and CoOOH, Co_2O_3 as reference (d). The $FeCoO_x$ is a homogeneous phase of bimetallic hydroxide, not the single-phase Fe or Co hydroxides.

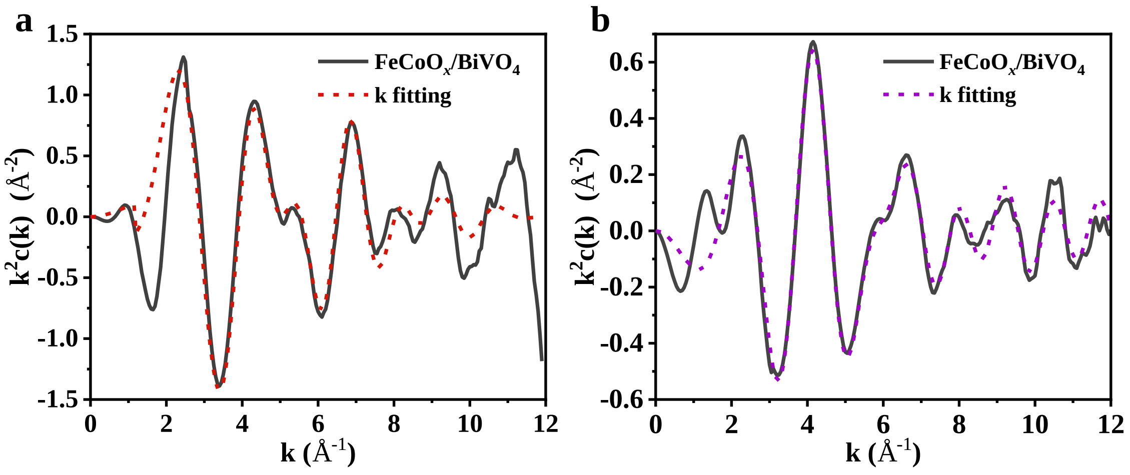

Supplementary Fig. 9 $k^2\chi(k)$ space spectra fitting curves of $FeCoO_x/BiVO_4$. (a) Fe K-edge; (b) Co K-edge. The fitting of χ(k) space spectra is good.

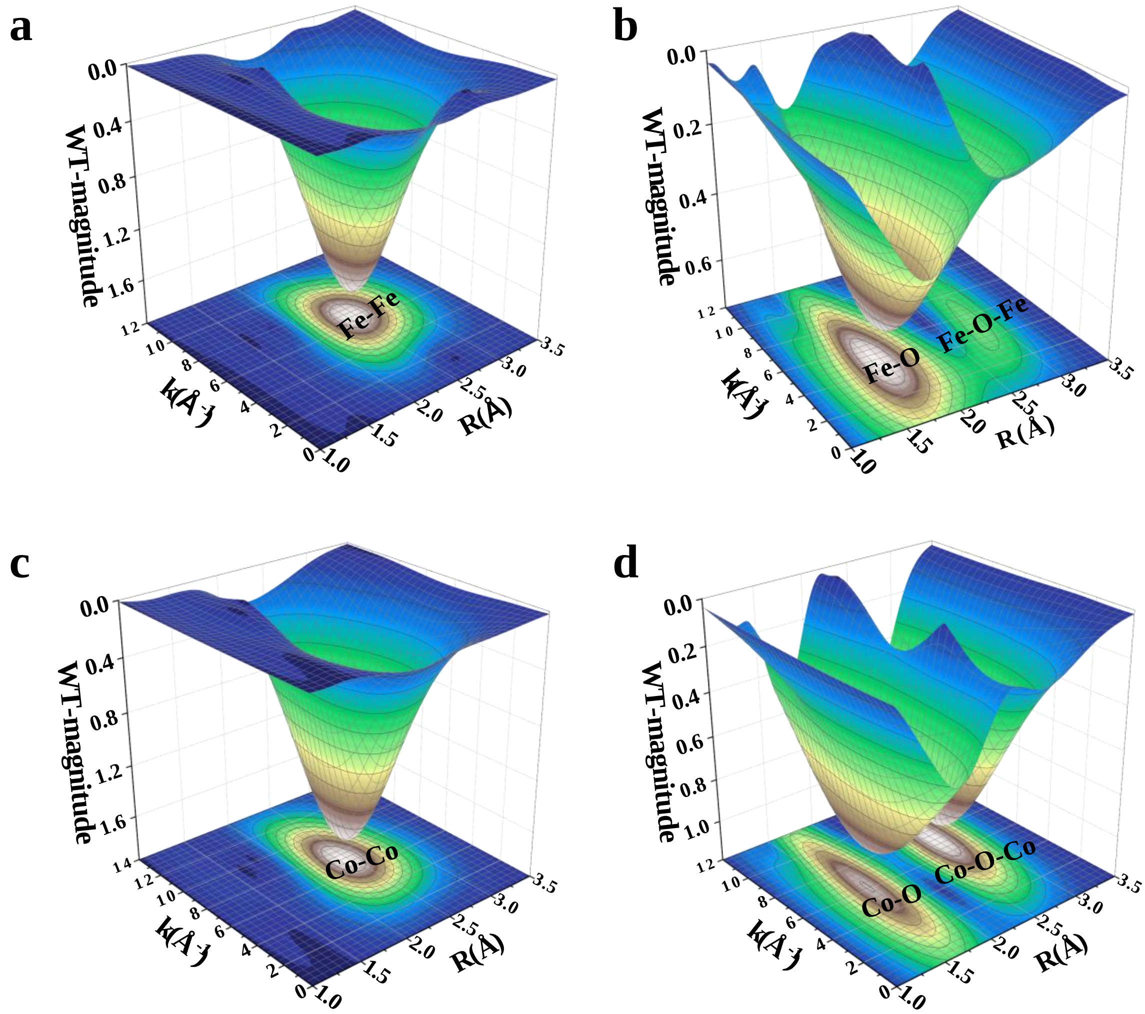

Supplementary Fig. 10 3D contour Wavelet of reference samples. The extended X-ray absorption fine structure (WTEXAFS) map with 2D projection of Fe K-edge of Fe foil (a) and FeOOH (b), Co K-edge of Co foil (c) and CoOOH (d), respectively. There are no Fe-Fe, Co-Co, Fe-O-Fe and Co-O-Co bonds in the $FeCoO_x/BiVO_4$ sample after comparing with the references.

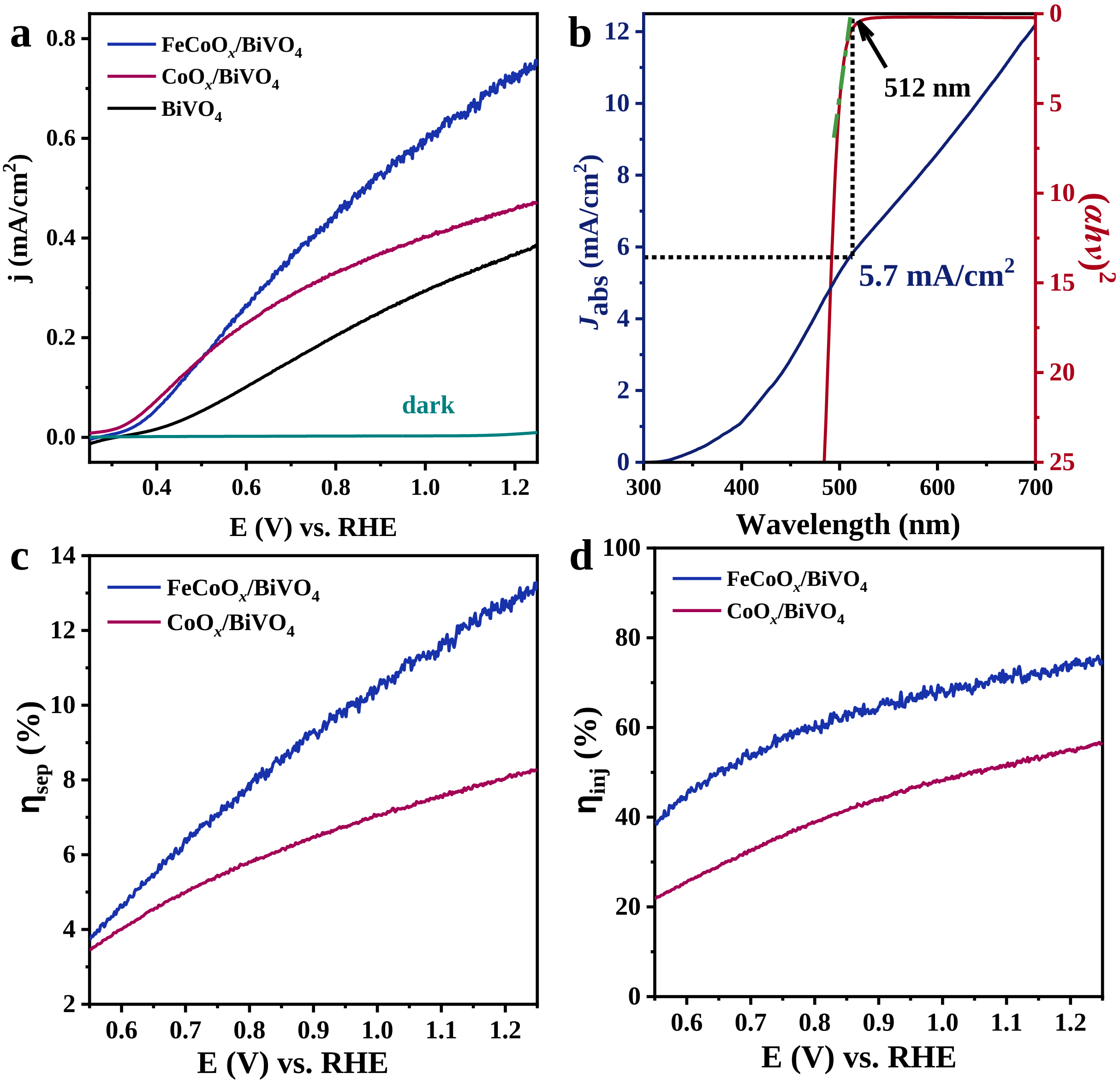

Supplementary Fig. 11 Photoelectrochemical performance and analysis of FeCoO$_x$/BiVO$_4$ and CoO$_x$/BiVO$_4$. (a) The photocurrent-voltage (J-V) curves of FeCoO$_x$/BiVO$_4$ and CoO$_x$/BiVO$_4$ in the electrolyte containing 1 M potassium borate (KBi, pH 9) and 0.2 M Na_2SO_3.

(b) The integrated maximum photocurrent (j_{abs}) of BiVO$_4$ photoanodes based on the UV-vis DRS spectra.

(c) Charge injection efficiencies of CoO$_x$/BiVO$_4$ and FeCoO$_x$/BiVO$_4$ at different bias voltages.

(d) Charge separation efficiencies of CoO$_x$/BiVO$_4$ and FeCoO$_x$/BiVO$_4$ at different bias voltages.

The J-V curves of the typical photoanodes in the presence of 0.2 M Na_2SO_3 (hole scavenger, $j_{sulfite}$) are given in Supplementary Fig. 11a. According to the equations: $\eta_{inj} = j_{water}/j_{sulfite}$ and $\eta_{sep} = j_{sulfite}/j_{abs}$, where j_{abs} is the maximum photocurrent density that a photoanode can achieve (jabs = 5.7 mA/cm^2 determined by the light absorption of $BiVO_4$, Supplementary Fig. 11b).

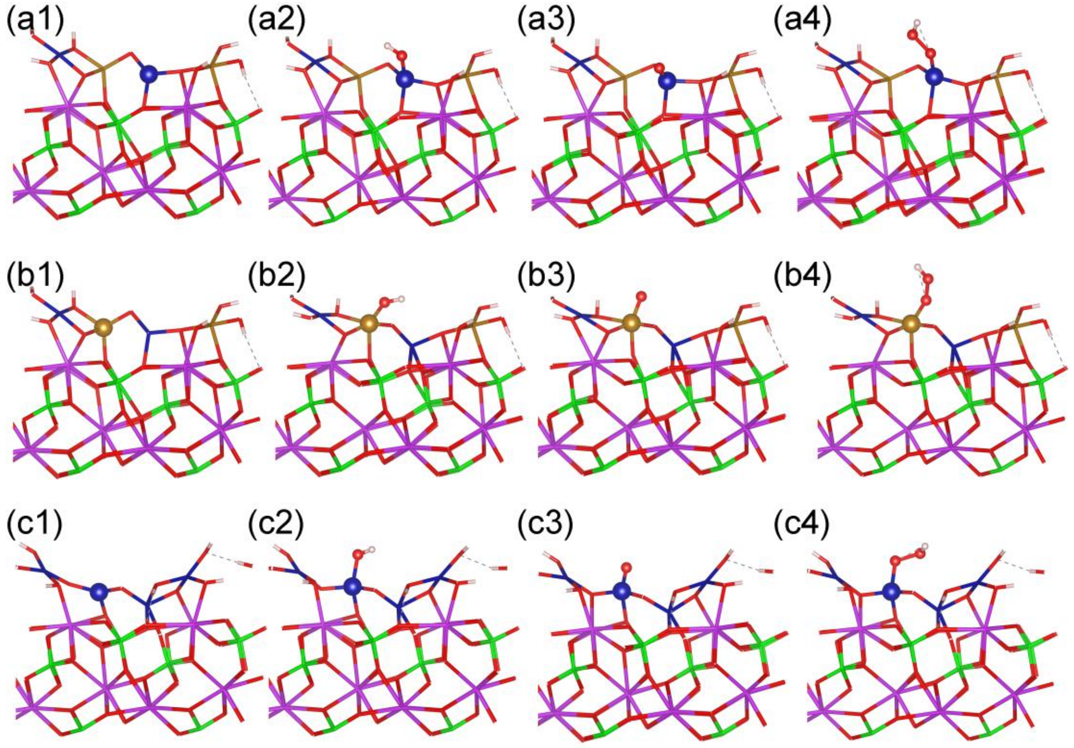

Supplementary Fig. 12 Schematic of the whole OER mechanism on the $FeCoO_x/BiVO_4$ and $CoO_x/BiVO_4$. (a1) to (a4) denote *, OH^*, O^*, OOH^* of Co site of $FeCoO_x/BiVO_4$. (b1) to (b4) denote *, OH^*, O^*, OOH^* of Fe site of $FeCoO_x/BiVO_4$. (c1) to (c4) denote *, OH^*, O^*, OOH^* of Co site of $CoO_x/BiVO_4$, respectively.

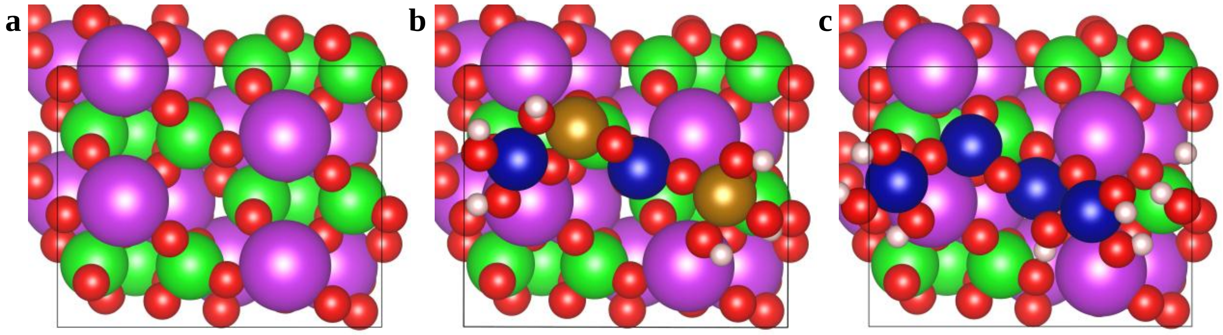

Supplementary Fig. 13 Bird view of the interface structures for DFT calculations. (a) $BiVO_4$ {110} surface, (b) $FeCoO_x/BiVO_4$ {110} interface and (c) $CoO_x/BiVO_4$ {110} interface.

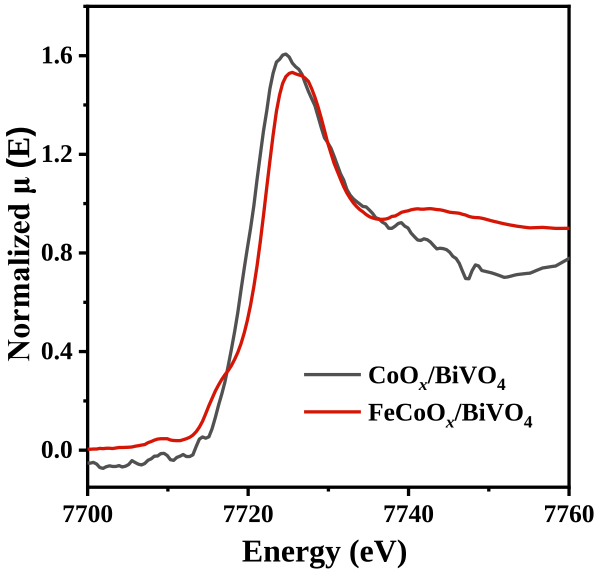

Supplementary Fig. 14 Structural characterizations of typical samples. The Co K-edge XANES μ(E) spectra of the $CoO_x/BiVO_4$ and $FeCoO_x/BiVO_4$. The valence state of Co in $FeCoO_x/BiVO_4$ is higher that of $CoO_x/BiVO_4$, which is consistent with the changing trend of bader charge from the DFT calculations.

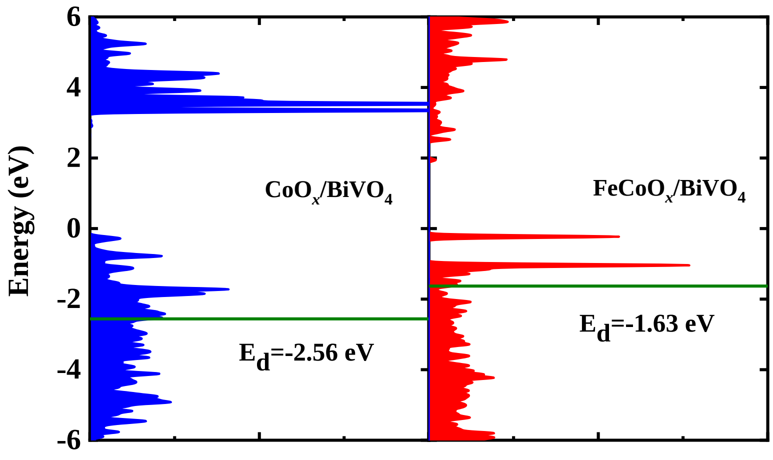

Supplementary Fig. 15 Comparison of d-band center (Ed) of active sites. The Ed value of Co sites in FeCoO$_x$/BiVO$_4$ and CoO$_x$/BiVO$_4$. The d-band center (Ed) value of Co active sites in FeCoO$_x$/BiVO$_4$ is calculated as -1.63 eV, and the Co active sites in CoO$_x$/BiVO$_4$ is -2.56 eV.

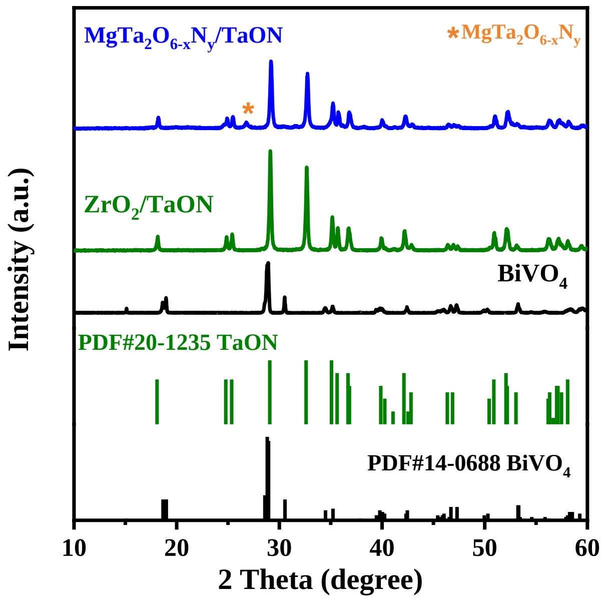

Supplementary Fig. 16 Structure characterization of the HEP and OEP. The XRD patterns of MgTa$_2$O$_{6-x}$N$_y$/TaON, ZrO$_2$/TaON and BiVO$_4$. The synthesized MgTa$_2$O$_{6-x}$N$_y$/TaON, ZrO$_2$/TaON and BiVO$_4$ all well with their respective PDF cards.

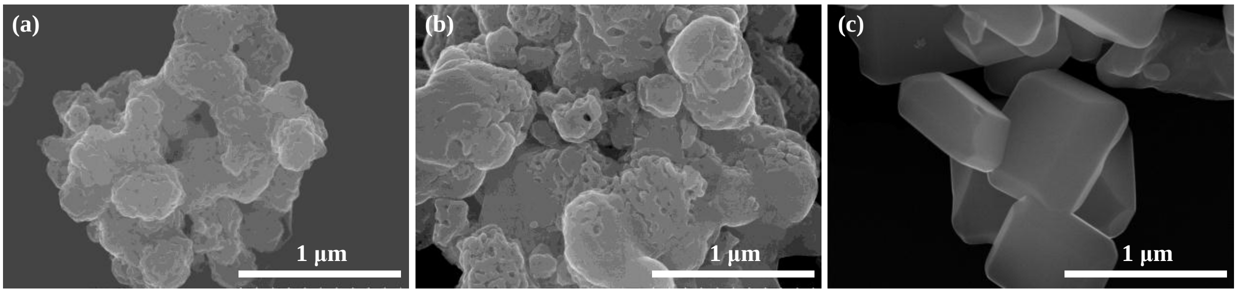

Supplementary Fig. 17 Typical FESEM images of the HEP and OEP. (a) ZrO_2/TaON, (b) $MgTa_2O_{6-x}N_y$/TaON and (c) $BiVO_4$. The surface of ZrO_2/TaON is rough and porous, while the surface of $MgTa_2O_{6-x}N_y$ is smooth. The $BiVO_4$ is a decahedron structure with exposed {010} and {110} facets.

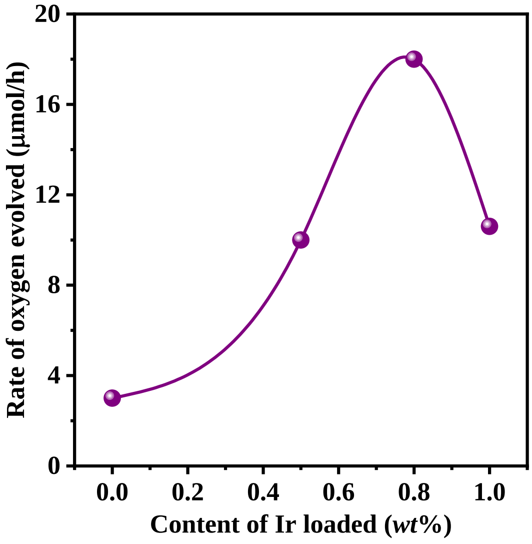

Supplementary Fig. 18 The rates of O_2 evolution on the Ir/$BiVO_4$ as a function of Ir content. The optimal loading content of Ir is 0.8 *wt*%.

Reaction conditions: 20 mg photocatalyst; 20 mL 50 mM sodium phosphate buffer solution (pH 6.0) containing $K_3[Fe(CN)_6]$ (5 mM); 300 W xenon lamp ($\lambda \geq 420$ nm), 0.5 h top-irradiation.

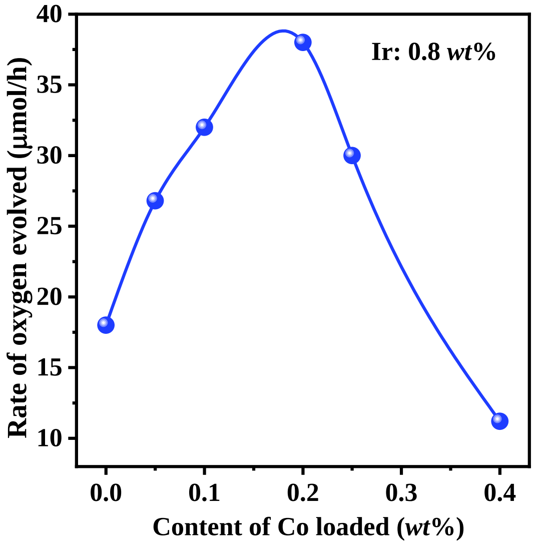

Supplementary Fig. 19 Optimization of the Co loading amounts with regard to photocatalytic O_2-evolving rates. The optimal content of Co is 0.2 *wt*%.

Reaction conditions: 20 mg $BiVO_4$ (0.8 *wt*%Ir fixed); 20 mL 50 mM sodium phosphate buffer solution (pH 6.0) containing $K_3[Fe(CN)_6]$ (5 mM); 300 W xenon lamp (λ ≥ 420 nm), 0.5 h top-irradiation.

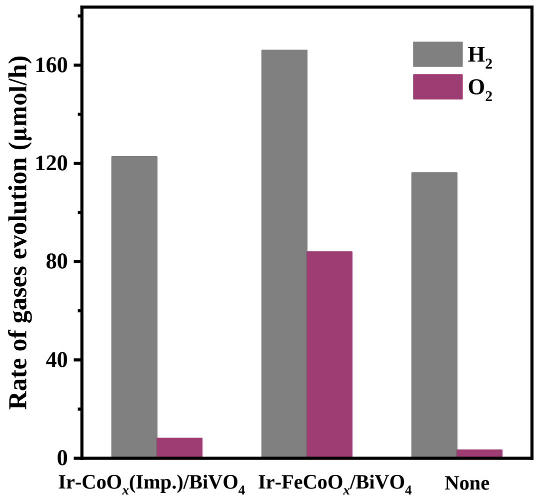

Supplementary Fig. 20 Comparison of the Z-scheme OWS performances using $BiVO_4$ without and with dual-cocatalysts loaded by different methods. When using the Ir-CoO_x(Imp.)/$BiVO_4$ with Ir and Co randomly dispersion or $BiVO_4$ as the OEP, the OWS will not be achieved.

Reaction conditions: 50 mg $BiVO_4$ with dual-cocatalysts loaded using different methods, 50 mg HEP (ZrO_2/TaON, 1.0 *wt*% Rh, 1.5 *wt*% Cr), 100 mL 25 mM sodium phosphate buffer solution (pH 6.0) containing $K_4[Fe(CN)_6]$ (10 mM), 300 W xenon lamp (λ ≥ 420 nm), temperature: 298 K, Pyrex top-irradiation type.

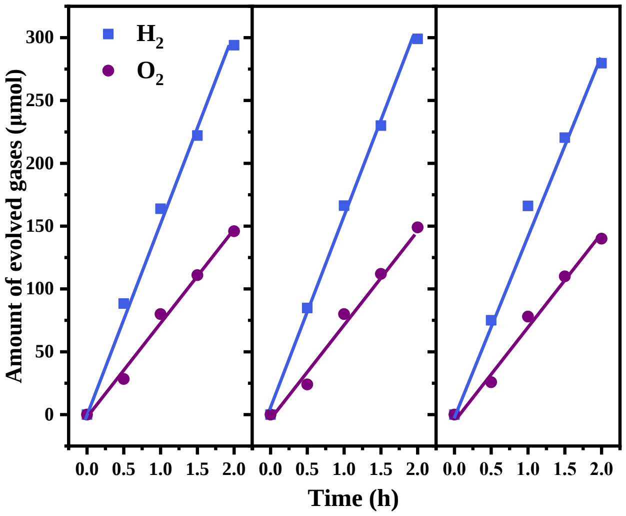

Supplementary Fig. 21 Multiple cycles of Z-scheme overall water splitting using Ir-FeCoO$_x$/BiVO$_4$ as the OEP and Rh$_y$Cr$_{2-y}$O$_3$-ZrO$_2$/TaON as the HEP via [Fe(CN)$_6$]$^{3-/4-}$ redox mediator. After the vacuum treatment, the activity does not decrease, indicating the good photostability.

Reaction conditions: 50 mg OEP (0.8 *wt*% Ir; 0.2 *wt*% Co), 50 mg HEP (1.0 *wt*% Rh, 1.5 *wt*% Cr), 100 mL 25 mM sodium phosphate buffer solution (PBS pH = 6.0) containing $K_4[Fe(CN)_6]$ (10 mM), 300 W xenon lamp ($\lambda \geq 420$ nm), temperature: 288 K, Pyrex top-irradiation type.

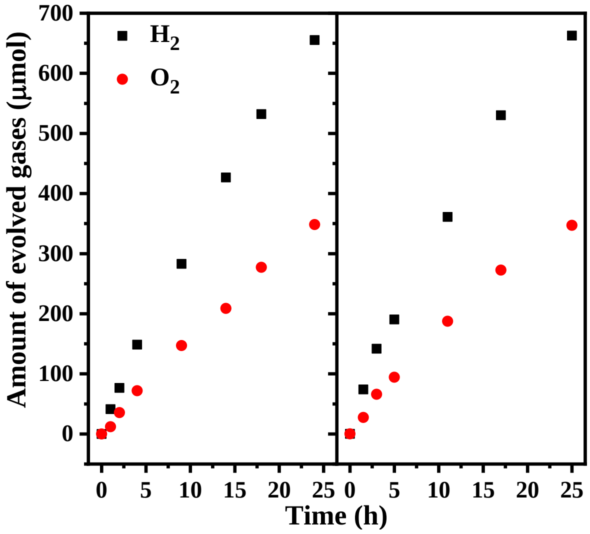

Supplementary Fig. 22 Multiple cycles of curve of Z-scheme OWS under illumination of the standard solar simulator (AM 1.5G, 100 mW cm^{-2}). The activity maintains well after long time irradiation, indicating the good photostability of the system.

Reaction conditions: 50 mg OEP (0.8 *wt*% Ir; 0.2 *wt*% Co), 50 mg HEP (ZrO$_2$/TaON, 1.0 *wt*% Rh, 1.5 *wt*% Cr), 100 mL 25 mM sodium phosphate buffer solution (pH 6.0) containing $K_4[Fe(CN)_6]$ (10 mM), temperature: 288 K, Pyrex top-irradiation type.

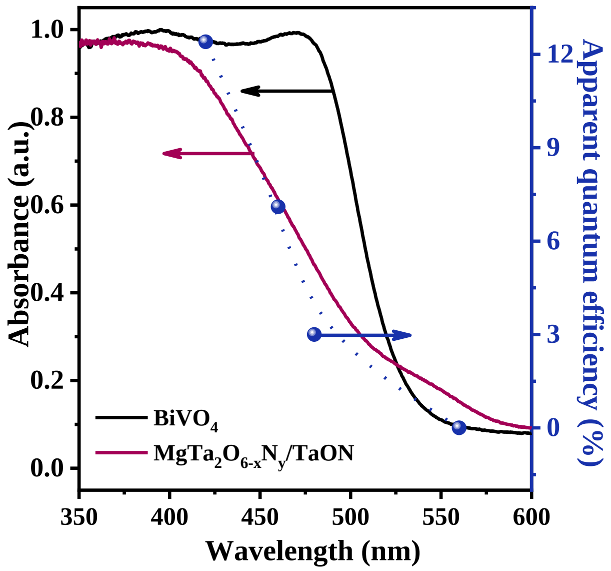

Supplementary Fig. 23 Dependence curve of AQE value as a function of irradiation wavelength and UV-vis DRS of the HEP and OEP. The AQE value of OWS is in good accordance with the UV-vis DRS of $MgTa_2O_{6-x}N_y$/TaON and $BiVO_4$ as a function of absorption wavelength.

Reaction conditions: 75 mg OEP (Ir-$FeCoO_x$/$BiVO_4$, 0.8 *wt*% Ir; 0.2 *wt*% Co), 150 mg HEP ($MgTa_2O_{6-x}N_y$/TaON, 2.5 *wt*% Rh, 3.75 *wt*% Cr), 150 mL 25 mM sodium phosphate buffer solution (pH 6.0) containing $K_4[Fe(CN)_6]$ (10 mM), 300 W xenon lamp, temperature: 298 K, Pyrex top-irradiation type.

Supplementary Tables

Supplementary Table 1 Structural parameters extracted from the K-edge Fe and Co $\chi(R)$ space spectra fitting of $FeCoO_x/BiVO_4$

	Reduced Chi-square (χ_v^2)	R-factor (%)
Fe K-edge $FeCoO_x/BiVO_4$	9831.42	0.0524

amp/ S_0^2	$N_{(Fe\text{-}O\ path)}$	$R_{(Fe\text{-}O\ path)}$ (Å)	$\sigma^2_{(Fe\text{-}O\ path)}$ (10^{-3} Å^2)	ΔE_0 (eV)
0.96+/-0.16	4.5	1.884 ± 0.064	4.9+/-1.8	5.08+/- 2.49
amp/ S_0^2	$N_{(Fe\text{-}O\text{-}Co\ path)}$	$R_{(Fe\text{-}O\text{-}Co\ path)}$ (Å)	$\sigma^2_{(Fe\text{-}O\text{-}Co\ path)}$ (10^{-3} Å^2)	ΔE_0 (eV)
1.06 +/-0.12	2	2.745 ± 0.103	6.2+/-2.5	6.30+/-2.78

	Reduced Chi-square (χ_v^2)	R-factor (%)
Co K-edge $FeCoO_x/BiVO_4$	4604.98	0.0290

amp/ S_0^2	$N_{(Co\text{-}O\ path)}$	$R_{(Co\text{-}O\ path)}$ (Å)	$\sigma^2_{(Co\text{-}O\ path)}$ (10^{-3} Å^2)	ΔE_0 (eV)
0.94+/-0.12	3.5	1.756 ± 0.023	4.9+/-1.8	3.41+/- 1.72
amp/ S_0^2	$N_{(Co\text{-}O\text{-}Fe\ path)}$	$R_{(Co\text{-}O\text{-}Fe\ path)}$ (Å)	$\sigma^2_{(Co\text{-}O\text{-}Fe\ path)}$ (10^{-3} Å^2)	ΔE_0 (eV)
0.98 +/-0.13	2	2.761 ± 0.037	6.2+/-2.5	5.55+/-2.44

Supplementary Table 2 Values of R_s and R_{ct} of various $BiVO_4$-based electrodes loaded with different reduction cocatalysts

Entry	Photocatalysts	R_s (Ohm)	R_{ct} (Ohm)
1	$BiVO_4$/FTO	38.7	5156
2	Au/$BiVO_4$/FTO	58.9	1123
3	Ir/$BiVO_4$/FTO	32.9	473

Supplementary Table 3 Values of R_s and R_{ct} of various $BiVO_4$-based electrodes loaded with different oxidation cocatalysts

Entry	Photocatalysts	R_s (Ohm)	R_{ct} (Ohm)
1	$BiVO_4$/FTO	49.2	3976
2	CoO_x/$BiVO_4$/FTO	48.4	2552
3	$FeCoO_x$/$BiVO_4$/FTO	45.1	1878

Supplementary Table 4 Calculated Bader charge for Fe, Co within the CoO_x/$BiVO_4$ and $FeCoO_x$/$BiVO_4$

	Co in CoO_x/$BiVO_4$	Co in $FeCoO_x$/$BiVO_4$	Fe in $FeCoO_x$/$BiVO_4$
Bader charge	1.2 a.u.	1.3 a.u.	1.6 a.u.

Supplementary Table 5 Free energy corrections for species.

Species	ZPE	TΔS
H_2O	0.573	0.67<br>(0.035 bar)
H_2	0.284	0.403
O*	0.066	0.092
OH*	0.359	0.110
OOH*	0.427	0.168

Supplementary Table 6 Comparison of details of ΔG_1, ΔG_2, ΔG_3, ΔG_4, and overpotential of water oxidation on the $FeCoO_x/BiVO_4$ and $CoO_x/BiVO_4$.

Index	ΔG_1	ΔG_2	ΔG_3	ΔG_4	η
$FeCoO_x/BiVO_4$ Co site	0.304	1.413	1.657	1.546	0.427
$FeCoO_x/BiVO_4$ Fe site	-0.071	1.445	2.079	1.467	0.849
$CoO_x/BiVO_4$	1.083	1.074	1.789	0.974	0.559

Supplementary Methods

Materials and reagents

For the preparation of ZrO_2-modified TaON, $MgTa_2O_{6-x}N_y$/TaON composite and $BiVO_4$ samples, Ta_2O_5 (99.9%, High Purity Chemicals), $ZrO(NO_3)_2 \cdot 2H_2O$ (ZrO_2 45.0%, Guangfu Chemical Reagent), $MgSO_4$ (99.5%, Alfa Aesar), $Bi(NO_3)_3 \cdot 5H_2O$ (99.0%, Sinopharm Chemical), NH_4VO_3 (99.0%, Sinopharm Chemical) and polyvinyl alcohol (PVA, Sigma-Aldrich, 99%) were used. $Na_3RhCl_6 \cdot 12H_2O$ (Rh 17.1%, Alfa Aesar), K_2CrO_4 (99.5% Kermal Chemical Reagent), K_2IrCl_6 (Ir: 39% Alfa Aesar) and $CoSO_4 \cdot 7H_2O$ (99.5%, Sinopharm Chemical) were employed as the precursors of cocatalysts. Methanol (99.5%, Sinopharm Chemical) and $K_4[Fe(CN)_6] \cdot 3H_2O$ (99.5%, Sinopharm Chemical) were used as hole acceptors. $K_3[Fe(CN)_6]$ (99.5%, Sinopharm Chemical) and $NaIO_3$ (99.8%, Guangfu Chemical Reagent) were used as electron scavengers. All chemicals were used as-purchased without further purification.

Preparation of Ir-CoO_x(Imp.)/$BiVO_4$

Typically, 0.2 g $BiVO_4$ sample was immersed in a calculated K_2IrCl_6 (0.8 *wt*%) and $CoSO_4$ (0.2 *wt*%) aqueous solution with ultrasonic agitation for ca. 5 min. After the solution was completely evaporated in a water bath at 353 K, the impregnated powder was collected and calcinated at 673 K for 1 h under air. Afterwards, it was reduced at 573 K for 1 h under a flow of 5% H_2/Ar (200 mL min^{-1}) .

Characterizations of photocatalysts

X-ray diffraction (XRD) measurement was carried out on a Rigaku D/Max-2500/PC powder diffractometer (Cu Kα radiation) with an operating voltage of 40 kV and an operating current of 200 mA. The scan rate applied was 5° min^{-1} with a step size of 0.02° in the experimental range. UV-Vis diffuse reflectance spectra (DRS) was recorded on a UV-Vis spectrophotometer (JASCO

V-550) equipped with an integrating sphere, and $BaSO_4$ powder was used as the reference for baseline correction. The morphologies and particle sizes were examined by field emission scanning electron microscopy (FESEM; S-5500, Hitachi) taken with a Quanta 200 FEG scanning electron microscope. High-resolution transmission electron microscopy (HRTEM), scanning transmission electron microscopy (STEM), electron energy loss spectroscopy (EELS) analysis and element mapping were performed on a JEOL JEM-ARM200F microscope operating at 200 kV with an electron energy filter (Gif Quantum ER) as well as the X-ray electron energy dispersive spectrometer (EDS, JEOL Co.,). The lock-in-based surface photovoltage (SPV) spectroscopic measurement system consisted of a 500 W xenon lamp (LSH-X500, Zolix), a grating monochromator (Omni-5007, Zolix), a lock-in amplifer (SR830-DSP, Stanford) with a light chopper (SR540, Stanford), a photovoltaic cell, and a computer. Monochromatic light was provided by passing light from a 500 W xenon lamp through the grating monochromator. A low chopping frequency of 23 Hz was used in the conventional testing. And the measurements were carried out in air atmosphere at room temperature.

Photocatalytic reactions

The photocatalytic reactions were carried out in a Pyrex top irradiation-type reaction vessel connected to a closed gas circulation system. Before reactions, the mixed solution containing catalysts was evacuated and then irradiated using a 300 W Xenon lamp with a cut-off filter (Hoya, L-42; $\lambda \geq 420$ nm). A flow of cooling water was used to maintain the reaction system at 288 K. The gases evolved were analyzed by gas chromatography (Agilent; GC-7890A, MS-5A column, TCD, Ar carrier).

X-ray absorption fine structure (XAFS) measurements

The XAFS spectra of Fe K-edge were collected at BL14W1 beamline of Shanghai Synchrotron Radiation Facility (SSRF) while Co K-edge were collected at 1W1B beamline of Beijing

Synchrotron Radiation Facility (BSRF). The data were collected in fluorescence mode using a Lytle detector while the corresponding reference samples were collected in transmission mode. The samples were grinded and uniformly daubed on the special adhesive tape.

XAFS analysis and results

The acquired EXAFS data were processed according to the standard procedures using the ATHENA module of Demeter software packages.

The extended X-ray absorption fine structure (EXAFS) spectra were obtained by subtracting the post-edge background from the overall absorption and then normalizing it with respect to the edge-jump step. Subsequently, the χ(k) data were Fourier transformed to real (R) space using a hanning windows (dk=1.0 $Å^{-1}$) to separate the EXAFS contribution from different coordination shells. To obtain the quantitative structural parameters around central atoms, least-squares curve parameter fitting was performed using the ARTEMIS module of Demeter software packages

The following EXAFS equation was used:

$$\chi(k) = \sum_j \frac{N_j S_0^2 F_j(k)}{k R_j^2} \cdot \exp[-2k^2\sigma_j^2] \cdot \exp[\frac{-2R_j}{\lambda(k)}] \cdot \sin[2kR_j + \phi_j(k)]$$

the theoretical scattering amplitudes, phase shifts and the photoelectron mean free path for all paths calculated. S_0^2 is the amplitude reduction factor, $F_j(k)$ is the effective curved-wave backscattering amplitude, N_j is the number of neighbors in the jth atomic shell, R_j is the distance between the X-ray absorbing central atom and the atoms in the jth atomic shell (backscatterer), λ is the mean free path in Å, $\phi_j(k)$ is the phase shift (including the phase shift for each shell and the total central atom phase shift), σ_j is the Debye-Waller parameter of the jth atomic shell (variation of distances around the average R_j). The functions $F_j(k)$, λ and $\phi_j(k)$ were calculated with the ab initio code FEFF9. The additional details for EXAFS simulations are given below.

All fits were performed in the *R* space with *k*-weight of 2 while phase correction was also

applied in the first coordination shell to make R value close to the physical interatomic distance between the absorber and shell scatterer. The coordination numbers of model samples were fixed as the nominal values. The obtained S_0^2 was fixed in the subsequent fitting. While the internal atomic distances R, Debye-Waller factor σ^2, and the edge-energy shift Δ were allowed to run freely.

Computational details

The density-functional theory (DFT)[1,2] calculations with the Hubbard U (DFT+U) corrections were performed with the Vienna Ab-initio Simulation Package (VASP)[3] codes 5.4. The PAW[4,5] pseudo-potentials, as well as the Perdew-Burke-Ernzerhof[6] exchange-correlation functional, and a plane wave cutoff of 500 eV have been used in the calculations. For a better description of the 3d electrons, the hybrid HF-DFT PBE0 functional is employed in our calculations[7]. All periodic slab calculations were carried out using a vacuum spacing of at least 12 Ang. All surface structural models had a minimum 7 Ang thickness with 2 Ang thickness are fixed. 2x2x1 Monkhorst-Pack k-point mesh is sampled in the calculations. Spin-polarized calculations were identified for all surfaces, and the convergence of energy and force was set to 10^{-5} eV and 0.03 eV/angstrom. The DFT-D3 method[8,9] was adopted to consider van der Waals correction.

Theoretical Evaluation of Activity

The theoretical overpotentials for $FeCoO_x/BiVO_4$ and $CoO_x/BiVO_4$ interfaces were determined, assuming the conventional oxygen evolution reaction (OER) mechanism[10]. The computational hydrogen electrode model[11] was used for the expression of the chemical potentials of protons and electrons at any given pH and applied potential as in previous OER works[12,13]. The elementary steps by which the OER occurs are believed to involve adsorbed OH, O and OOH species on the surface (*) according to the following equations:

$$OH^- + ^* \rightarrow OH^* + e^- \quad (1)$$

$$OH^* + OH^- \rightarrow O^* + H_2O + e^- \quad (2)$$

$O^* + OH^- \rightarrow OOH^* + e^-$ (3)

$OOH^* + OH^- \rightarrow ^* + O_2 + H_2O + e^-$ (4)

To model the thermochemistry of the OER, it is more convenient to work at acidic condition, where steps 1, 2, 3, and 4 are modified as:

$H_2O + ^* \rightarrow OH^* + H^+ + e^-$(5)

$OH^* + H_2O \rightarrow O^* + H_2O + H^+ + e^-$ (6)

$O^* + H_2O \rightarrow OOH^* + H^+ + e^-$ (7)

$OOH^* + H_2O \rightarrow ^* + O_2 + H_2O + H^+ + e^-$(8)

Thus, the Gibbs free energy change for steps 5 to 8 can be expressed as:

$\Delta G_1 = \Delta G_{OH} - eU + \Delta G_{H+}(pH)$ (9)

$\Delta G_2 = \Delta G_O - \Delta G_{OH} - eU + \Delta G_{H+}(pH)$ (10)

$\Delta G_3 = \Delta G_{OOH} - \Delta G_O - eU + \Delta G_{H+}(pH)$ (11)

$\Delta G_4 = 4.92eV - \Delta G_{OOH} - eU + \Delta G_{H+}(pH)$ (12)

Here, -eU accounts for the applied external bias U; ΔG_{H+}(pH) = −kBTln (10) × pH is the free energy change due to a nonzero pH value; The sum of ΔG_{1to4} is fixed to the negative of experimental Gibbs free energy of formation of two H_2O molecules 4.92 eV in order to avoid the calculation of the O_2 bond energy, which is difficult to accurately determine within DFT calculations. The Gibbs free energy differences of these intermediates include zero-point energy (ZPE) and entropy corrections. Specifically, the entropic contributions from H_2 and H_2O molecules are taken from the experimental results and the contributions from absorbed species on the surface are all listed in Supplementary Table 5. The theoretical overpotential is then be defined as: $\eta = max(\Delta G_1, \Delta G_2, \Delta G_3, \Delta G_4)/e - 1.23eV$ (13). The comparison of details of ΔG_1, ΔG_2, ΔG_3, ΔG_4, and overpotential of $FeCoO_x/BiVO_4$ and $CoO_x/BiVO_4$ are listed in Supplementary Table 6.

Supplementary References

1. Hohenberg, P., Kohn, W. Inhomogeneous electron gas. *Phys. Rev.* **136**, B864 (1964).

2. Kohn, W., Sham, L. J. Self-consistent equations including exchange and correlation effects. *Phys. Rev.* **140**, A1133 (1965).

3. Kresse, G., Furthmüller, J. Efficient iterative schemes for ab initio total-energy calculations using a plane-wave basis set. *Phys. Rev. B* **54**, 11169 (1996).

4. Blöchl, P. E. Projector augmented-wave method. *Phys. Rev. B* **50**, 17953 (1994).

5. Kresse, G., Joubert, D. From ultrasoft pseudopotentials to the projector augmented-wave method. *Phys. Rev. B* **59**, 1758 (1999).

6. Perdew, J. P., Burke, K. & Ernzerhof, M. Generalized gradient approximation made simple. *Phys. Rev. Lett.* **77**, 3865 (1996).

7. Adamo, C., Barone, V. Toward reliable density functional methods without adjustable parameters: The PBE0 model. *J. Chem. Phys.* **110**, 6158 (1999).

8. Grimme, S., Ehrlich, S. & Goerigk, L. Effect of the damping function in dispersion corrected density functional theory. *J. Comput. Chem.* **32**, 1456–1465 (2011).

9. Moellmann, J.; Grimme, S. DFT-D3 study of some molecular crystals. *J. Phys.Chem. C* **118**, 7615–7621 (2014).

10. Rossmeisl, J., Logadottir, A. & Nørskov, J. K. Electrolysis of water on (oxidized) metal sur- faces. *Chem.Phy.* **319**, 178–184 (2005).

11. Nørskov, J. K. et al. Origin of the overpotential for oxygen reduction at a fuel-cell cathode. *J. Phys. Chem. B* **108**, 17886–17892 (2004).

12. Bajdich, M. et al. Theoretical investigation of the activity of cobalt oxides for the electrochemical oxidation of water. *J. Am. Chem. Soc.* **135**, 13521–13530 (2013).

13. Seitz, L. C. et al. A highly active and stable $IrO_x/SrIrO_3$ catalyst for the oxygen evolution reaction. *Science* **353**, 1011–1014 (2016).
